# Strengthening counseling on barriers to exclusive breastfeeding through use of job aids in Nampula, Mozambique

**DOI:** 10.1371/journal.pone.0224939

**Published:** 2019-12-02

**Authors:** Justine A. Kavle, Melanie Picolo, Gabriela Buccini, Iracema Barros, Chloe H. Dillaway, Rafael Pérez-Escamilla

**Affiliations:** 1 United States Agency for International Development (USAID)’s Maternal and Child Survival Program (MCSP) / PATH, Washington, District of Columbia, United States of America; 2 USAID’s Maternal and Child Survival Program (MCSP) / PATH, Maputo, Mozambique; 3 Department of Social and Behavioral Science, School of Public Health, Yale University, New Haven, Connecticut, United States of America; Medical Research Council, SOUTH AFRICA

## Abstract

**Introduction:**

While the Government of Mozambique has galvanized action around exclusive breastfeeding (EBF) as a national priority, only 43% of Mozambican children under six months of age are exclusively breastfed. In the absence of skilled lactation support, challenges mothers experience with breastfeeding may inhibit initiation, exclusivity and duration. There is insufficient evidence on how to strengthen health providers’ competencies to address breastfeeding challenges in low- and middle-income countries. The objectives of this study were to 1) assess EBF challenges, from the perspectives of health providers and mothers; 2) ascertain the quality of health provider counseling to address EBF challenges; and 3) gain an understanding of the usefulness of job aids to improve counseling within routine health contact points in Nampula, Mozambique.

**Methods:**

This implementation science study was conducted in Meconta and Mogovolas districts, Nampula province, Mozambique from July-November 2018. In Phase 1, 46 in-depth interviews with mothers and providers, and 11 observations of counseling sessions were conducted. In Phase 2, health providers were trained to use three job aids (i.e., facility, community or maternity contacts) to identify and address EBF problems during routine health services. In Phase 3, 30 in-depth interviews with mothers and providers were conducted to assess the experience with job aid use. In both Phase 1 and 3, we conducted a thematic analysis using a grounded theory approach involving a step-wise coding process.

**Results:**

Poor latch and positioning, perceived insufficient breastmilk and breast engorgement emerged as barriers to EBF. Providers often lacked the knowledge, skillset, and self-efficacy to manage EBF problems, with little counseling provided at community or facility levels. Following job aid rollout, providers reported improved assessment of breastfeeding technique, and increased self-efficacy and motivation to identify and resolve EBF problems.

**Conclusions:**

Integration of job aids, with clear lactation management guidance, into maternal and child health training curricula and supportive supervision is critical to building providers’ skillsets and competencies to provide quality lactation counseling and support.

## Introduction

Optimal breastfeeding practices reduce neonatal and child morbidity (i.e., respiratory infection and diarrhea) and mortality, and have demonstrated protective effects against obesity and diabetes [1–5]. Exclusive breastfeeding (EBF) is defined as the proportion of infants aged zero to five months who are fed only with breastmilk and no additional liquids or solids until six months of life [6]. In the last two decades, progress in EBF has been limited, as only 41% of infants are exclusively breastfed in low and middle income countries [7]. Lactating women may experience challenges to maintaining EBF for the first six months of an infant’s life, as demonstrated in a recent systematic review which identified sixteen barriers to EBF including maternal perceptions of insufficient breastmilk, early introduction of foods and liquids prior to six months of age, and lack of counseling on physical breast problems. Yet, these barriers are often not adequately addressed through infant and young child feeding (IYCF) programs and initiatives, and further, are not reflected in country investments and access to skilled lactation support [8,9].

In Mozambique, EBF practices are suboptimal. According to 2011 DHS data, about three-quarters of infants initiate breastfeeding within the first hour of life, while only 43% of infants under six months are exclusively breastfed. About half of infants are introduced to food and/or liquids other than breastmilk prior to three months of age. According to a qualitative formative assessment using Trials of Improved Practices (TIPs) methodology conducted in Angoche and Malema districts, which are considered to be representative of cultural and geographic differences that influence maternal and infant and young child nutrition practices in Nampula Province, some mothers were advised by health professionals to wait to offer the breast to the infant, at times, up to three to four days after birth [10]. Mothers discussed discarding colostrum and early introduction of foods prior to six months of age as a remedy to breastmilk insufficiency due to non-receipt of provider advice on colostrum, and lack of information on the value of breastfeeding and timely introduction of complementary foods [10]. In 2011, a qualitative study in Mozambique reported that infants were frequently introduced to traditional medicines, water, and porridges prior to six months of age [11]. Moreover, most health workers lacked skills to adequately counsel and address breastfeeding challenges that mothers experienced.

The Government of Mozambique has galvanized action around EBF as a national priority. The 2019 National Infant and Young Child Feeding Strategy set targets for EBF, for children zero to five months of age, to increase from 43% (2011) to 55% by 2023. The Multisectoral Action Plan for Stunting Reduction and The National IYCF policy and strategies recommend key actions for EBF including training health workers [namely community-based personnel, such as traditional birth attendants and community health workers (Agente Polivalente Elementar)] within the health system; distributing EBF information, education and communication materials; creating private breastfeeding spaces (“*breastfeeding corners*”) in health facilities; implementing the Baby Friendly Hospital Initiative (BFHI); establishing community mother support groups; and regular monitoring of the implementation of the National Code for the Marketing of Breastmilk Substitutes.

Barriers and challenges to skilled lactation care and counseling can hinder early initiation, exclusivity, and duration of EBF, which is a key component of nutrition and health programming in Mozambique. Yet, an important gap exists in the literature on the minimum health provider competencies required to address breastfeeding challenges, in terms of clinical and practical skills as well as types of training delivery in low and middle income countries, as highlighted in recent WHO guidance [12]. To address this gap, the objectives of this study were to:

1. Identify problems and challenges with EBF experienced by mothers in rural and semi-urban areas in Nampula, Mozambique.
2. Gain an understanding of mothers’ care-seeking patterns for addressing the identified breastfeeding problems and challenges.
3. Gain an understanding of the quality and type of counseling on breastfeeding problems and challenges currently provided by facility and community-based health providers, and how providers can improve counseling on EBF through the use of a job aid at delivery, postpartum and community routine health contact points.
4. Assess the usefulness of job aids to improve the successful identification of and counseling on barriers to EBF by facility- and community-based health providers, and identify ways to improve them, through their rollout and use within existing service delivery entry points.

## Methods

### Program background

This study was implemented within the context of the Maternal and Child Survival Program (MCSP), a global, 5-year cooperative agreement funded by the United States Agency for International Development (USAID) to introduce and support scale-up of high-impact health interventions among USAID’s 25 maternal and child health priority countries, as well as other countries [13]. In Mozambique, MCSP worked with the Ministry of Health (MOH) in Nampula and Sofala provinces to improve the quality of preventative and curative nutrition programming, including infant and young child counseling from May 2017 to January 2019.

### Ethics statement

Potential study participants were led through the verbal informed consent process by the study team member within the privacy of participant’s home (or space near the home) or health facility which provided auditory privacy to ensure confidentiality. The informed consent process encompassed all the study details in an understandable language (i.e., Portuguese or Macua based on participant’s preference) to the participant regarding the study and the fact that his/her participation would be completely voluntary. All study participants provided verbal informed consent for voluntary participation in the study, following a description of the study’s purpose, and granted permission to audio record the interviews. Verbal informed consent was documented by signature of the interviewer (as a witness) and a copy of the consent form was given to each participant. This study received Human Subjects Review approval from National Committee of Bioethics for Health in Mozambique, PATH, and Yale University’s Human Subjects Institutional Review Boards.

### Study design and sites

This implementation science study was conducted in two districts, Meconta and Mogovolas, in Nampula Province, Mozambique. The study sites were selected due to existence of community structures for nutrition programming, existence of MCSP project activities, and physical accessibility. These sites are also representative of geographic and cultural differences with regards to breastfeeding practices in Nampula Province and may be generalized to other rural areas in northern Mozambique. Within each district, two health facilities were purposively selected to recruit participants (mothers and providers) for in-depth interviews (IDIs) and observation and included the main health facility in each district and a peripheral health facility that was accessibly located.

**Fig 1:**
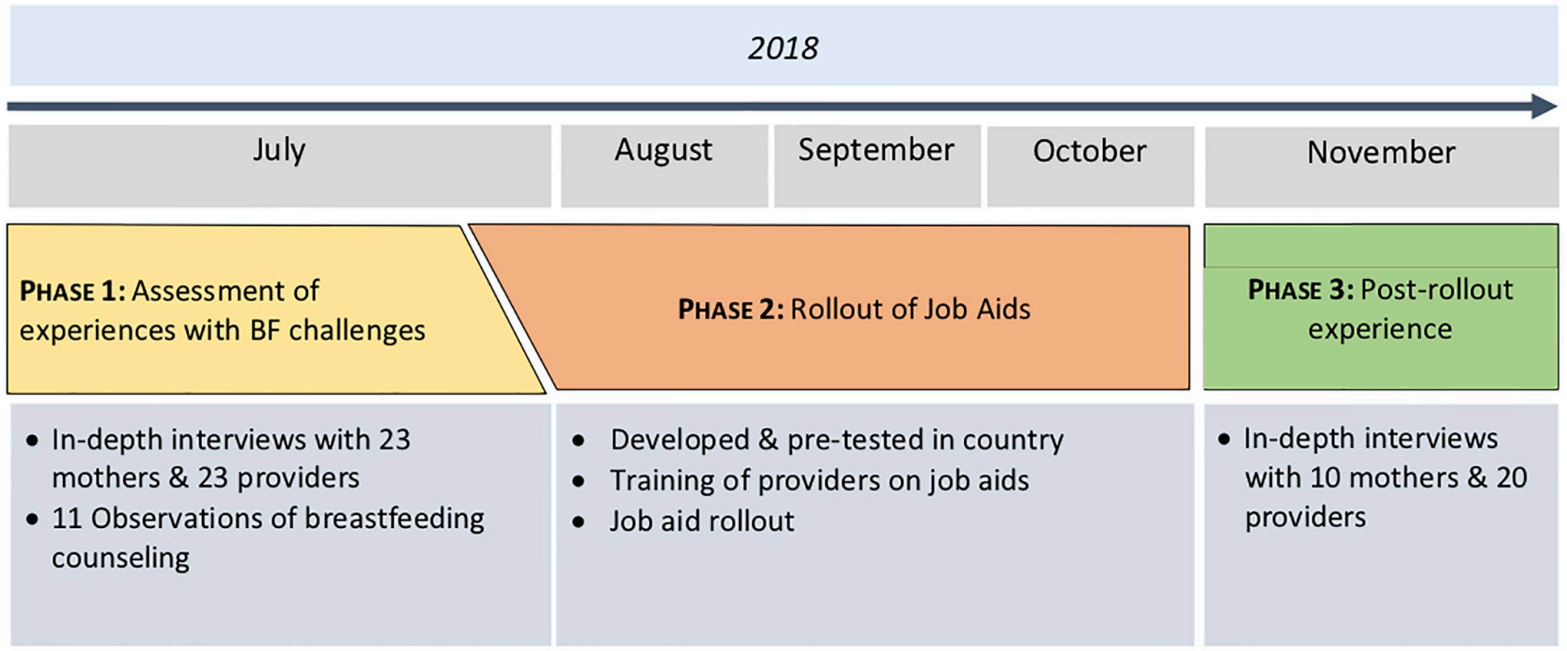
Phases 1, 2 & 3: Summary of study components.

This study was conducted over three phases (Fig 1). In Phase 1, we aimed to assess mothers’ experiences with EBF challenges and the support and counseling provided by facility- and community-based health providers to address these challenges by conducting a first round of semi-structured IDIs and observations of breastfeeding counseling sessions. In Phase 2, job aids to assist facility- and community-based providers in identifying and addressing common EBF problems were developed and piloted in Mozambique. Health providers were trained on job aid use. In Phase 3, following a three-month rollout of the job aids, IDIs were conducted to determine the usefulness of the job aids in improving providers’ identification of and counseling for barriers to EBF. Suggestions to improve the job aids from health providers and mothers were collected. Details of each phase are provided in Fig 1.

### Study participant selection

#### In-depth interviews with providers and lactating mothers

Using purposive sampling, for Phase 1 and 3 IDIs, lactating mothers (i.e., defined as mothers who were currently breastfeeding) of infants zero to five months were eligible provided they 1) were residents of the selected study sites in Nampula province; 2) were at least 18 years of age; and 3) had received counseling from a community or facility-based health provider who used a study job aid (Phase 3 only). Facility- and community-based health providers were eligible provided that they 1) worked in Nampula province and within MCSP-supported facilities and communities; 2) provided nutrition and child health services; and 3) in Phase 3 only, had been trained on and used a study job aid. Mothers were excluded from Phase 1 and Phase 3 if they had 1) infants born with less than 35 weeks of gestation and/or 2) infants with any chronic illness or disability that would markedly affect normal feeding practices.

#### Observation of breastfeeding counseling sessions

As part of Phase 1, direct observations of individual (mother-provider) breastfeeding counseling sessions were conducted to ascertain the type and quality of breastfeeding counseling skills of facility health providers. On the day of data collection, the team worked with health providers to approach and purposively select mothers during postnatal care, growth monitoring or other routine services at the health facility while they waited in line to be seen. Mothers were screened for eligibility, based on whether mothers were currently breastfeeding and attending routine health facility services, according to Phase 1 eligibility criteria listed above.

### Data collection

All study materials, including IDI guides, observation guides and job aids, were designed based on a review of barriers to EBF and associated literature from low- and middle-income countries [8,16,17] and were pre-tested and adapted to the local context prior to implementation. The study team conducted the pre-test in the District of Rapale, Nampula Province, a rural area, similar in profile to other study sites, which was selected due to its vicinity to the Provincial Capital of Nampula City. Two IDIs with health facility providers were conducted in Portuguese and one focus group discussion with community health workers, lactating women, and male partners, was conducted in the local language of Macua, to determine the study participants comprehension of terminology used in the data collection tools. The results of the pre-test were used to make small adjustments in the data collection tools. The sample size for each study phase was determined based on the principle of data saturation.

#### In-depth interviews with providers and lactating mothers

Phase 1 IDIs were conducted in July 2018 by local interviewers trained in qualitative research methods and EBF counseling and Phase 3 IDIs were conducted in November 2018 by trained MCSP nutritionists. All but one local interviewer had previous experience in the maternal and child nutrition field. All interviewers were trained on confidentiality, how to conduct qualitative in-depth interviews, and on key concepts of breastfeeding counseling. During data collection in phase 1, at the end of each day, a meeting was held to debrief about each interview, including preliminary findings, questions, and perceptions about the process and information collected. Although different participants were interviewed for each phase (phases 1 and 3) demographic characteristics of the two samples were similar. Community-based health providers and mothers´ interviews were conducted in the local language, Macua, and facility-based health providers’ interviews were conducted in Portuguese. Interviews were conducted in each participant’s place of work or at the mother’s home and lasted between 15 and 40 minutes. A team of native Portuguese and Macua speakers transcribed the interviews verbatim in Portuguese. In a small number of cases, audio recordings were not available and interview notes were used in lieu of full transcripts.

#### Observation of breastfeeding counseling sessions

Observations of breastfeeding counseling sessions were conducted by GB, an International Board-Certified Lactation Consultant (IBCLC). Breastfeeding counseling was conducted by the health provider in Macua and a trained MCSP nutritionist carried out real-time translation to Portuguese for the observer. The sessions lasted between 1 minute and 14 minutes each. A breastfeeding counseling observation guide was used to assess the quality of breastfeeding counseling for each session, which included best practices for breastfeeding counseling established by WHO/UNICEF [17] (i.e., breastfeeding history, breastfeeding assessment, and breastfeeding counseling skills). The observation guide provided a checklist of breastfeeding counseling skills, which were marked by the observer if and when these skills were used by the provider, and it was supplemented by field notes. The use of these best practices for effective BF counseling, delineated in the checklist, was considered ‘high quality of breastfeeding counseling’.

### Job aid development and rollout

Beginning in April 2017, MCSP worked with the Nutrition Technical Working Group in Nampula Province to develop three job aids (1) at childbirth, for use either at the health facility or community levels; (2) at postnatal and child health visits at health facility level; and (3) at community contact points during the first 6 months of the postnatal period. The job aids were developed in a flowchart format to guide health providers through a step-by-step process to (1) support mothers to use and practice various breastfeeding techniques for optimal latch and positioning and prevent potential breastfeeding problems; (2) assess if the mother uses appropriate breastfeeding techniques; (3) identify and resolve common breastfeeding problems; and (4) advise on when to refer breastfeeding problems to facility level (community-based providers only). Illustrations aided health providers in each step, which are aligned with the MOH Mozambique IYCF counseling materials.

In July 2018, 10 facility-based health providers, 17 community-based health providers, and four district level maternal and child health and nutrition focal points in the study sites were trained on use of the job aids. Following the trainings, suggestions from participants and facilitators on how to improve the materials were incorporated in the final versions. In August 2018, the materials were distributed to trained providers along with a registry of all breastfeeding women with infants under six months of age who were counseled by providers with the job aids at routine contact points in MCSP study areas. MCSP district nutrition officers provided routine supportive supervision and mentoring on the use of the job aids during the rollout period (August-October 2018). Health providers who were not exposed to the job aids, often due to facility staff turnover, were trained by MCSP district nutrition officers during the rollout period.

### Data analyses

#### In-depth interviews with providers and lactating mothers

In both Phase 1 and 3, we conducted a thematic analysis using a grounded theory approach involving a step-wise coding process adapted from Bradley et al [18]. We analyzed transcripts based on a predetermined structure to answer our research questions (described above) in each phase. First, for Phase 1, two investigators (GB, RPE) independently read transcripts and then each investigator individually open-coded a subset of transcripts, assigning labels representing ideas in the text. Next, the research team (GB, RPE, JAK, MP and IB) established consensus on code usage, definitions, and structure and developed an initial codebook. The remainder of the transcripts were then coded by one investigator (GB) using the codebook. Finally, the coding of interviews and codebook was refined iteratively with the research team (GB, RPE, JAK, MP and IB), who provided input by reading a subset of transcripts to ensure agreement on code organization and definition. A similar process was carried out for Phase III, with a subset of the investigators (JAK, MP and IB) independently reading transcripts, establishing consensus on code usage and definitions, and coding transcripts. The transcripts were coded and classified using Dedoose v.8.1.8 qualitative computer software, applying codes in English to the original Portuguese transcripts. Conceptual themes and subthemes were generated throughout the coding process based on research questions. We identified quotes that best represented the themes and translated them from Portuguese into English for reporting purposes. To assess the change in health provider knowledge, counseling skills, and motivation between phases 1 and 3, we collated quotes from each identified domain, and categorized whether demonstration of knowledge, skills or attitudes was (1) “lacking” (defined as few to no providers), (2) “somewhat infrequent” (defined as some but not all providers) or (3) “consistent” (defined as nearly all providers).

#### Observation of breastfeeding counseling sessions

We conducted qualitative analysis of counseling observation data by analyzing field notes of each session according to the breastfeeding counseling best practices and counseling skills outlined in the WHO/UNICEF framework [17]. Health providers were assessed based on whether they demonstrated each of the skills outlined in the framework, including taking breastfeeding history, assessing breastfeeding, communicating in a supportive manner, building confidence, and providing practical help.

## Results

### Routine contact points in the health system

EBF support and counseling occurs through multiple routine contacts, at childbirth, postnatal care, and child health services in the Mozambique health system, as described in Table 1.

**Table 1:** Breastfeeding counseling and support at routine contact points within the Mozambique health system.

**Antenatal Care**	
**One-on-one counseling**	During antenatal care visits, nutritional counseling is often limited to maternal diet. The opportunity for counseling on early initiation of breastfeeding and appropriate breastfeeding techniques, and for providing encouragement or making a plan to ensure EBF for six months is missed.
**Group talks**	Each day, before consultations begin, maternal and child health nurses often provide group health education sessions on a range of topics, including family planning, care during pregnancy, breastfeeding, hygiene and sanitation, among others. As observed in Phase 1, group education sessions focusing on breastfeeding seldom included practical support for breastfeeding or use of job aids to explain crucial breastfeeding techniques and how to manage common breastfeeding challenges and problems.
**Maternity services**	
**48-hour hospital stay**	Maternity registers indicate that most mothers put their babies to the breast within one hour after birth. During routine visits to maternity wards conducted by MCSP staff, one-on-one counseling and practical support were rarely observed.
**Group talks**	Daily group education sessions are an opportunity to provide new mothers with information on newborn care and feeding, so that all mothers have the essential information they need before discharge. Counseling on breastfeeding focuses on the promotion of EBF for the first six months of life. Some practical support may be provided to mothers facing difficulties.
**Postnatal care and child visits**	
**Group breastfeeding promotion talks**	Breastfeeding talks are the most common breastfeeding promotion activity at the health facility level. Breastfeeding talks aim to motivate mothers to exclusively breastfeed for six months. While the talks were not designed to solve or manage breastfeeding problems, breastfeeding promotion activities at the community and health facility levels were cited as an opportunity to strengthen EBF counseling.
**Child health visits**	Every day prior to the start of well child visits (CCS), mothers are invited to participate in educational talks that last about 10 minutes, with a different topic covered each day, including EBF. Children under five years of age are seen during CCS for growth monitoring (weight and height assessment), vaccination, micronutrient supplementation beginning at six months, and deworming (at one year of age). In the first year of life, children are seen monthly and if the child is growing well with no problems diagnosed, they are seen for bi-monthly consultations after one year of age. While mothers reported participating in lectures on breastfeeding during CCS, they did not report receiving individual advice on breastfeeding during CCS consultations.

#### Routine contact points outside the health system

The National Institute of Social Action (Instituto Nacional de Acção Social [INAS]) implements social assistance programs to individuals who are unable to meet their basic needs either temporarily or permanently. The Direct Social Support Program–Child Kit (PASD-B) was designed to support households who are in need of breastmilk substitute because they are in recovery from acute malnutrition, or due to the mother or child’s inability to breastfeed. Eligibility for PASD-B includes (1) a child less than 24 months of age, (2) a medical referral for a breastmilk substitute due to the child’s clinical condition, or ii) a medical referral for a breastmilk substitute due to the mother’s clinical condition, or iii) presentation of a death certificate for the mother (which is often the case with caregivers of orphans).

### Phase 1: Pre-job aid rollout findings

Participant characteristics from Phase 1 IDIs are described in Table 2. The majority of mothers were less than 26 years of age, had less than a primary school education, and worked as farmers. Facility-based providers were younger than community-based providers and had between one and seven years of work experience, while community-based providers had between one and 31 years of experience. Facility-based providers included nutrition technicians, preventive medicine technicians and maternal and child health nurses. Community-based providers included activists (community health workers that are generally trained and may receive incentives, usually paid through projects at the community level), traditional birth attendants, and a polyvalent agent (community health worker that is part of the national health system).

**Table 2:** Phase 1 and Phase 3 participant characteristics.

Participant Characteristic	In-depth Interviews		Observations
	Phase 1	Phase 3	Phase 1
**Mothers**	n = 23	n = 10	n = 11
Age			
18–19	4	3	0
20–24	13	3	9
26–40	6	4	2
Education			
No formal education	5	1	2
Some primary	10	5	6
Primary completed	4	4	1
Some secondary	4	0	0
Secondary completed	0	0	2
Marital status ^a^			
Married or living together	20	10	11
Divorced	1	0	0
Work status			
Not working outside the home (domestic work and farming)	7	9	4
Working outside the home (working in the field or other commercial activity)	14	1	7
Maternity leave	2	0	0
Infant accompanies to work ^b^			
Yes	11	0	7
No	1	0	0
N/A (mother does not work outside home / is on maternity leave)	8	9	4
Infant age			
1–2 months	10	3	4
3–5 months	13	7	7
Infant sex ^c^			
Female	12	5	5
Male	10	5	6
**Facility-based providers**	n = 10	n = 10	n = 6
Age			
23–29	5	7	1
30–44	5	2	4
45–77	0	1	1
Gender			
Female	8	7	4
Male	2	3	2
Education			
Some primary school	0	1	0
Some secondary school	0	0	1
Completed secondary school	10	9	5
Position			
Nutritionist	2	1	2
Preventive medicine technician	3	1	2
General medicine technician	0	4	0
Nurse	5	3	2
Midwife	0	1	0
Years of experience			
1–2 years	3	7	2
3–10 years	7	2	4
10–36 years	0	1	0
**Community-based providers**	n = 13	n = 10	n/a
Age ^d^			
23–29	2	1	
30–44	7	5	
45–77	3	3	
Gender			
Female	6	5	
Male	7	5	
Education ^e^			
No formal education	2	0	
Some primary school	4	2	
Completed primary school	2	2	
Some secondary school	4	4	
Completed secondary school	1	0	
Position ^f^			
Activist	8	6	
Polyvalent agent	1	1	
Traditional practitioner / birth attendant	4	2	
Years of experience ^g^			
1–2 years	1	2	
3–10 years	8	6	
10–31 years	3	1	

^a^ 2 not reported in Phase 1 IDIs

^b^ 3 not reported in Phase 1 and 1 in Phase 3 IDIs

^c^ 1 not reported in Phase 1 IDIs

^d^ 1 not reported in Phase 3 IDIs

^e^ 2 not reported in Phase 3 IDIs

^f^ 1 not reported in Phase 3 IDIs

^g^ 1 not reported in Phase 1 and 1 in Phase 3 IDIs

We identified three major themes from Phase 1, prior to rollout of the job aids: (1) common challenges that impede EBF from perspectives of mothers and health providers, (2) type and quality of breastfeeding counseling and support at the community and facility level, and (3) provider training, skills and self-efficacy to address breastfeeding difficulties.

#### Common challenges that impede EBF from the perspectives of mothers and health providers

Knowledge and beliefs about EBF were not consistent. Mothers and health providers unanimously knew global recommendations for EBF for the first six months of life:

*[Exclusive breastfeeding] is breastfeeding until [the infant] turns six months*, *and I heard this in the hospital that I should give only breast milk until the infant completes six months of life and then introduce water and porridge*

–Mother, Meconta

Mothers and community-based health providers believed that during the first two days (i.e., prior to the onset of lactation) some mothers do not produce any breastmilk. Health providers and mothers do not have information on lactation physiology and therefore do not understand delayed onset of lactation.

*[During the first two days after the baby was born] I breastfeed anyway*, *he sucked and did not find anything until the next day that the milk began to come out*

–Mother, Mogovolas

Concerns surrounding insufficient milk continue until the infant reaches three and four months of age due to the perception that the infant was *thirsty and hungry* and must be satiated to sleep, which prompted early introduction of foods and liquids other than breastmilk, such as porridge and water, prior to six months of age.

*Some say*, *my baby is nursing a lot*, *he is hungry*, *he gets weak*, *so to avoid it I have to give my son something because then he goes to sleep*, *he fills up and I can stay an hour or two without the baby waking up*.

–Facility-based provider, Mogovolas

Improving maternal diet was reported as the primary way to address perceived insufficient breastmilk. Mothers and facility- and community-based health providers relayed that mothers who followed a *healthy diet* and consumed fresh cassava, peanuts, beans, and fresh vegetables would increase breastmilk production. Health providers advised mothers to eat certain foods that are believed to help increase breastmilk production.

*I had a baby and because I stayed for one day without having milk I was advised to eat peanuts*, *cassava*, *and beans to stimulate the milk let down*. *When mothers know that they do not have enough milk they must eat a lot*, *(…) as long as they are healthy and eat the recommended foods for producing enough milk for a child*, *the [mother’s] body itself helps for this [milk production] to happen*

–Community-based health provider, Mogovolas

Health providers mentioned referral of mothers with reported breastfeeding insufficiency to the INAS PASD-B public program to receive infant formula. Infant formula was recommended to resolve reported breastmilk insufficiency, regardless if infants were considered eligible for the program.

*There are cases where the mother no longer produces enough milk and when we know that this mother is not producing enough [breast]milk we advise her to practice mix feeding*. *Some mothers can buy [formulas] and others can’t*. *For these ones we provide them with a written referral to ‘Acção Social’ along with the statement of the community leader and then she starts receiving milk [infant formula]*.

–Facility-based health provider, Meconta

Latching problems (e.g., baby not latching properly and sore nipples), and breast engorgement (e.g., swollen breasts) emerged as key barriers to early initiation of breastfeeding within the first days of life, as described by community and facility-based providers. As illustrated by one health provider:

*The majority of mothers have problems in the first days after giving birth at the beginning of breastfeeding (*…*) there have been mothers who have a swollen breast and this causes pain because the baby cannot suck all the milk (*…*) there are other women who have cracked nipple problems*

–Community-based health provider, Meconta

Mothers’ return to fieldwork early postpartum, as many mothers worked as farmers, also emerged as a challenge for most mothers. Infants were 2.8 months of age, on average, when mothers returned to work. Some mothers reported strategies to breastfeed their infants while in the field, such as either taking the infant to the fields or bringing a person (childminder or older sibling) to care for the child while the mother is working, allowing her to breastfeed throughout the day. Some mothers discussed how the father was involved as a principal source of support in caring for the infant.

*When I go to the farm I take [the child with me]*, *when I go to pick up nuts I leave with the father (*…*) If I leave the child with the father I express the milk from my breast and place it in a glass cup covered [for the father to offer the breastmilk in case the child cries]*.

–Mother, Meconta.

When a working mother does not have someone with whom to share the care of the infant, a few facility- and community-based providers mentioned the risk of early introduction of solids, bottle-feeding and pacifier use, which leave infants at risk of becoming acutely malnourished, which all interfere with EBF.

#### Type and quality of breastfeeding counseling and support at the community and facility level

**Fig 2:**
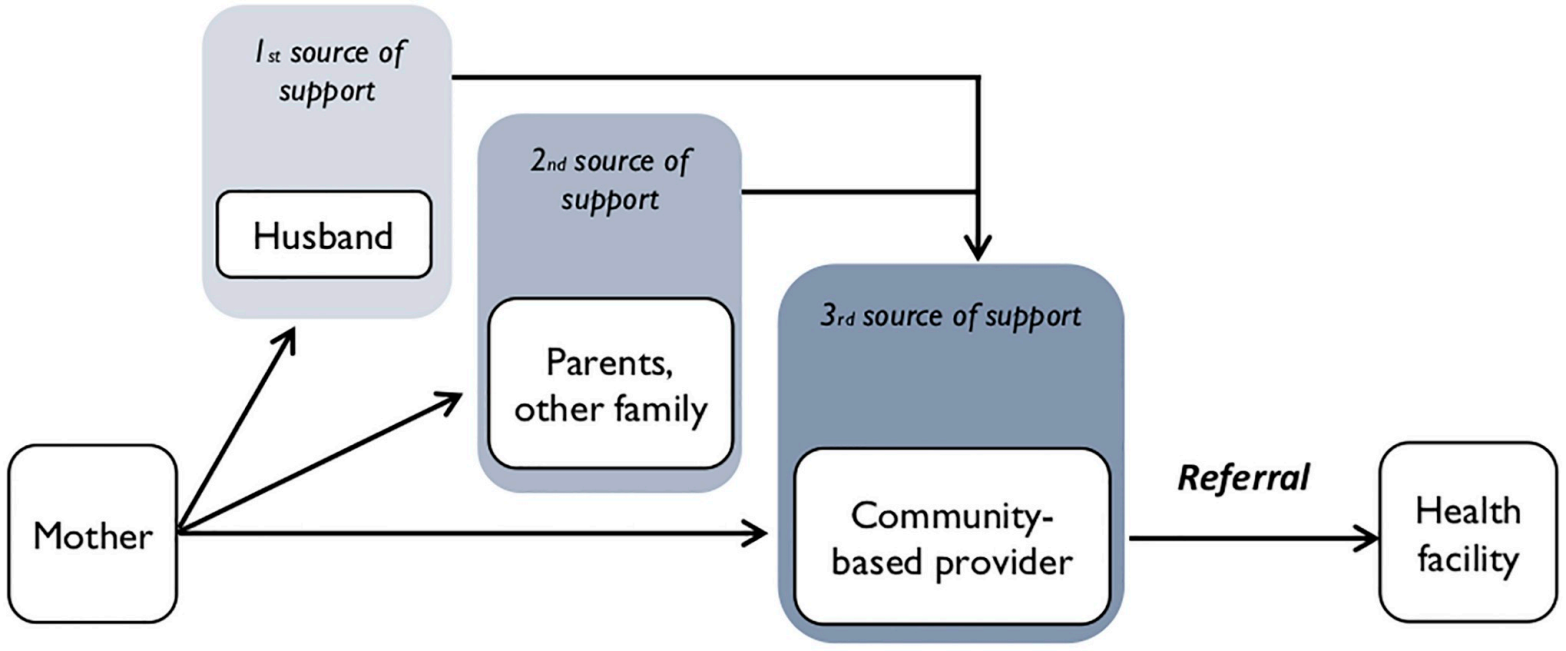
Sources of support for breastfeeding: Mothers’ perspectives.

At the community level, most mothers reported seeking help from husbands followed by parents/family members and community health providers (e.g. activists and traditional birth attendants) for EBF counseling in the first few months after delivery (see Fig 2).

Community health providers were the primary source of health care advice for mothers within the community when breastfeeding issues and challenges arise, and they mostly referred the mothers to the health facility if they are not able to help.

*[When a mother has a breastfeeding problem] she goes to the [community-based] activist because the activist worked with her during the pregnancy (*…*)*. *After the child has breastfeeding difficulties for one day or two days and the mother is already following what the activist recommended (…) [they] only refer to the health facility because there is no more information to give*, *the only option he has is refer to the health facility unit*.

–Community-based provider, Mogovolas

Facility-based health providers at the maternity ward reported providing counseling to mothers on breastfeeding positioning and skin-to-skin contact following delivery. During the first days after discharge, some mothers returned to the maternity ward due to early breastfeeding issues, such as difficulties with latching or delayed onset of lactation.

*When they are here [in the maternity ward] we explain that the baby sucks*, *but when they are back home*, *at the same day later they return to say that the baby no longer sucks and is crying a lot (*…*) and she says that the baby suckled here in the hospital but when the baby got home he/she did not breastfeed any more*, *he/she did not want to and we asked mother to explain how she breastfeed and we realized that she did not breastfeed well*.

–*Facility-based provider*, *Mogovolas*

While mothers reported participating in group lectures on breastfeeding during well child visits, they did not report receiving individual advice on breastfeeding during these consultations. Indeed, health providers often prioritized growth monitoring and immunization in the well child consultation despite counseling on breastfeeding difficulties. Individual breastfeeding counseling is provided only when a problem with weight gain was identified. In this situation, it was common for health providers to advise mothers regarding maternal diet during lactation.

*When a mother has a problem with her baby’s weight*, *I give advice right here [during the weighing and immunization]*, *I explain what she has to do (…) In exclusive breastfeeding*, *the mother should eat well to produce enough milk to feed her baby*. *Because she can produce enough milk until 6 months*, *but if she does not eat well she runs the risk of her breast getting dry*.

–Facility-based provider, Mogovolas

#### Health provider training, skills, and self-efficacy to address breastfeeding difficulties

The provision of practical support to address issues with breastfeeding positioning, latching, and other breastfeeding difficulties was not consistently reported. Health providers did not receive practical training in breastfeeding, signifying an important opportunity to strengthen breastfeeding counseling as part of the health care system. Facility-based health providers lacked pre-service training in breastfeeding skills and lactation topics. Providers reported receipt of training on counseling skills, which were not specifically related to breastfeeding. While breastfeeding counseling was generally not practiced in study sites, a few facility-based health providers recognized the importance of counseling skills for identifying and managing breastfeeding problems, as shown in the quotes below.

*Counseling in breastfeeding is to inform the mother first about what breast milk has*, *and why breast milk is important to give until 6 months*. *Counseling is an open conversation with no obligations*. *When I talk to the mothers and ask them to come in the next sessions*, *they come and that motivates me a lot*, *when I come and meet a larger number of mothers that motivates me*.

–Facility-based provider, Mogovolas

Most facility-based health providers had not received an in-service training on breastfeeding counseling. Most health providers discussed being able to identify that the mother was experiencing breastfeeding problems primarily through well child consultations, if the child did not gain weight during growth monitoring. Yet, the majority of providers lacked knowledge and self-efficacy to counsel on breastfeeding problems such as cracked nipples and physical breast problems.

*Cracked nipples*, *these are more frequent (*…*) [what is your advice for cracked nipples*?*] I advise to wash the wound and continue to offer the breast to the child (…) [why do you think these wounds happen*?*]*. *I do not know*.

–Facility-based provider, Meconta

On the other hand, while community-based health providers had received some training in breastfeeding counseling, they also lacked self-efficacy to manage breastfeeding problems, such as sore nipples or breast engorgement. Community-based health providers mostly identified problems and referred mothers to health facilities. Both facility and community-based health providers expressed their desire to receive continuous education trainings to update their knowledge and skills in handling breastfeeding problems.

*I wanted to know a lot more*! *Although I may have thought that poor milk production was because of this or that*, *if the mother is not producing milk*, *it could be a shortage of food*. *[Although] the mother can eat well*, *but there is another problem associated with this complication [poor milk production] beyond what I’m thinking*.

–Facility-based provider, Meconta

Health providers also mentioned work overload as an impediment to provision of EBF counseling. A high number of patients negatively impacted the quality of health talks, which included information on breastfeeding.

*There are days when we have a lot of work*, *a lot of patients (*…*) or we have urgencies that make it hard to stay in the talks for a long time*, *so we summarize it a bit*, *but we always give the talks*.

–Facility-based health provider, Mogovolas

Facility-based health providers also faced language barriers, as some providers do not speak the local language (Macua) and most mothers do not speak or read Portuguese. This may be a major barrier for EBF counseling since clear communication is essential for understanding and properly addressing breastfeeding issues. Another barrier that emerged was the low level of literacy among community-based health providers. These providers expressed difficulties in reading materials in Portuguese as well as remembering information given in a training in Portuguese.

### Phase 1 observation of breastfeeding counseling sessions

Eleven observations of breastfeeding counseling sessions were conducted with health facility providers. Eight observations were carried out during the well child consultations (growth monitoring and postnatal care) and three observations during the sick child consultations. Individual breastfeeding counseling sessions were uncommon in the designated study health facilities. It was noted that breastfeeding counseling was done only if the health provider was concerned with infant weight or growth. Health providers offered advice focused on the specific issue at hand; therefore, if a mother did not ask about breastfeeding, it was not addressed during her consultation.

Additionally, counseling sessions were very short in duration (one to fourteen minutes), which did not allow most health providers time to follow the best practices for effective EBF counseling (i.e., breastfeeding history, breastfeeding assessment, and breastfeeding counseling skills). Health facility providers provided counseling to build confidence and support, to reinforce mothers’ ability to breastfeed and provide care for the baby. Few providers asked for a history of the mother’s past or current breastfeeding challenges and experiences or assessed breastfeeding latch or positioning, even when a mother was breastfeeding during the consultation. Providers were observed to counsel mothers about the benefits of EBF and to not provide water or other food during the first six months as per the WHO recommendations or to provide advice if the infant was not gaining weight. None of the health providers gave practical support to manage breastfeeding problems.

### Phase 3 in-depth interviews

The majority of mothers were less than 25 years of age, had less than a primary school education, and were farmers (Table 2). Facility-based providers had completed secondary school, while community-based providers completed at most some secondary school education. Three themes emerged from the post-job aid interview on job aid use at facility and community level counseling: (1) job aid use and effect on counseling content and techniques for resolving breastfeeding problems; (2) effect of job aids on self-efficacy and motivation; and (3) improvements to job aids. The impact of the job aids on provider knowledge, skills and motivation is illustrated in Table 3.

**Table 3:** Knowledge, skills and motivation pre- and post- job aid rollout.

Job Aid Impact Domain		Phase 1: Pre-job aid rollout		Phase 3: Post-job aid rollout	
		Status	Illustrative quote	Status	Illustrative quote
**Provider knowledge on causes & management of breastfeeding problems**	Perceptions of insufficient breastmilk	F: **XX** C: **X**	There is a mother who said she did not produce milk, I advised her to eat vegetables to help produce milk, she followed my recommendations but still did not produce milk. I had to send her to Namitil where she could receive artificial milk because I had no solution.–Facility-based provider, Mogovolas	F: **XXX** C: **XXX**	Before when I saw a mother who had problems of not having [enough] milk, I advised only to eat certain foods … now after I received the material, I already have more knowledge … it is also great benefit to massage the breasts [to address insufficient breast milk].–Community-based provider, Mogovolas
	Engorgement & mastitis	F: **XX** C: **X**	*Those who have wounds and the breast gets full*, *I advise if the mother has conditions to prepare formula milk to give to the child or if the mother does not have money for formula here in our area we have a tradition of expressing coconut milk or peanut milk to give to the child*. - Community-based health provider, Mogovolas	F: **XXX** C: **XXX**	I have advised the mothers that if they have breast pain problems, tell me and I will guide you on how to overcome it. …If the breast is very full, until it hurts, I will advise you to extract the milk, not to throw it away.–Community-based provider, Meconta
	Cracked nipples	F: **XX** C: **X**	*[Mothers] say that they have wounds in their breast when the baby starts to breastfeed… and then they say that they cannot breastfeed otherwise they are going to transmit diseases (to the child)*. *I explain that if the problem is in one breast she can offer the other breast*, *because she cannot offer the one with problems because it can cause problems to the baby*.—Community-based health provider, Mogovolas	F: **XXX** C: **XXX**	Another situation the mother had a wound on the nipple and she was already giving up breastfeeding and I advised that she should give the breast. … Then I told her to go to the hospital. Also there [at the hospital] she was advised the same and came back and continued breastfeeding and the breast healed and she came back to thank me.–Community-based provider, Meconta
	Improper latch & positioning	F: **XX** C: **X**	*I only know how to give the mother a talk to give breast milk until 6 months*, *those techniques to get attached to the breast I did not know*.*—*Facility-based health provider, Mogovolas	F: **XXX** C: **XXX**	At first it was a difficult because I had no idea how they should hold the baby. After the training we had, I learned the proper techniques of breastfeeding. After the training, we can feel and notice that the milk is not reaching the baby. (…) First I start by checking the mother how she is holding the baby, if the areola is in her mouth and how the baby is sucking the milk.–Facility-based provider, Mogovolas
**Provider breastfeeding counseling skills**	Establish a dialogue / interactive counseling style	F: **X** C: **X**	In the health pre-service training, I learned that I have to allow space for questions and opinions while giving some advice, so mothers will be more willing [to receive advice]–Facility-based provider, Mogovolas	F: **XXX** C: **XXX**	First of all, after I introduced myself … From there I had to open the flipchart … [and] show it to the mothers. After showing it… I asked them what they were seeing in the pictures. I asked: how do you breastfeed your baby? They had to speak, and then from there [I asked]: if we’re looking at this mom here, what does she do?–Community-based provider, Mogovolas
	Provide practical support	F: **X** C: **X**	*(*…*) we have to teach how to breastfeed*, *the positioning that she should take to breastfeed the baby also counts a lot (*…*) I advise her to sit in a comfortable position and the baby should be skin contact with the mother’s body (*…*) I think most people breastfeed but they do not know these breastfeeding techniques*, *even the older mothers do not know (*…*) in these cases*, *I explain and the baby sucks and I say it was due to the position*, *I already had 3 cases and I taught*.*–*Facility-based provider, Mogovolas	F: **XXX** C: **XXX**	To demonstrate the latch… I watch each mother and see how the baby is doing the suction. I say, ‘this is correct’, if not I say, ‘you are breastfeeding, but it does not have to be in this way, it has to be this way.’ And also the mothers see those images [in the job aid], because first I have to do the talk with the job aid, then execute what is in the job aid.–Facility-based provider, Mogovolas
	CHW only: Manage breastfeeding problems prior to making a referral to the health facility	C: **X**	I would like a training to teach me how to help a mother with breastfeeding difficulties here in my community.–Community-based provider, Meconta	C: **XX**	Formerly I had no training and I soon referred to the health facility. … With swelling I give advice to breastfeed often in that breast that filled up. After 2 days, if the swelling does not go down, then I refer to the health facility.–Community-based provider, Mogovolas
**Provider Motivation**	Provider self-efficacy/ confidence in knowledge and skills to resolve breastfeeding problems	F: **X** C: **X**	*I did not give much advice I cannot lie*, *nor explained what to eat and how to breastfeed because we did not learn*, *I only give advice to the mother of what I was trained*.*–*Community-based provider, Meconta	F: **XXX** C: **XXX**	Now that I have this material that is very good, the information that I give is accurate (…) Now with this material, we talk and the mother can see the images that correspond to what we speak. (…) People used to hardly accept [our advice], but not today.–Community-based provider, Meconta
	Provider motivation to counsel on breastfeeding given response from mothers and perceived community impact	F: **X** C: **X**	It’s just that I do not know that it works a lot for those mothers, like doing mother-to-mother counseling, … and teach all of those [community-based] health providers … because they can greatly help change a lot in the behavior of the community itself because they are kings there and people obeyed them more than us [facility health providers], if they have knowledge it would greatly help.—Facility-based health provider, Mogovolas	F: **XXX** C: **XXX**	*From what I analyze*, *children are gaining weight because of the procedures (latching and positioning techniques) that mothers are using*. *I feel confident because the results of the children and the weight itself and the growth are showing a positive result compared to before*.*–*Community-based provider, Mogovolas

Demonstration of skill/knowledge/motivation as reported by providers in in-depth interviews

**X** Lacking/Infrequent

**XX** Somewhat frequent

**XXX** Consistent

F: Facility-based provider; C: Community-based provider

#### Job aid use and effect on counseling content and technique for resolving breastfeeding problems

Facility- and community-based providers incorporated the job aids into counseling in individual and group settings. Facility-based providers used the job aid across a variety of health contact points, including antenatal, postnatal, and adult visits, but most commonly during well child visits. The majority of community-based providers reported using the job aid only in group talks and home visits. Some community-based providers described using group talks to identify mothers with physical breast problems or incorrect breastfeeding technique(s), which included follow-up home visits to assist mothers in resolving any identified breastfeeding issues.

*I planned a day to counsel with this material*. *I called all mothers of children from zero to six months to advise [as a group]*. *(…) If today I gave the lecture and found a mother with difficulty*, *I had to register and then did home visits with mothers with difficulties two days later*. *For her to show me again what she has learned and [make sure she] is practicing [exclusive breastfeeding]*.

–Community-based provider, Mogovolas

Many facility-based providers reported that the job aid reaffirmed their existing knowledge about breastfeeding problems and served as a reminder of key topics. This translated into providers counseling on more topics during sessions, including how to position the baby and obtain a good latch. Some facility-based providers reported learning new information about breastfeeding technique from the job aid and others mentioned they learned about the expression and storage of breastmilk.

*I used to leave a lot of things aside that were really important*. *When I started using the material*, *I realized that many women did not know the right way [to breastfeed]*, *and this had many disadvantages for the children*. *… many mothers until this moment that we are talking know to position the baby well but do not know how to get a proper latch*.

–Facility-based provider, Mogovolas

Nearly all community-based providers reported that they learned new information about breastfeeding problems and technique from the job aid, which gave them the opportunity to disseminate new knowledge among community members. Other community-based providers mentioned that the job aid increased their knowledge about not introducing food or liquids besides breastmilk prior to six months.

*Before in the community the mothers … did not have the good latch*, *they only managed to give the breast to the child but they did not look at the procedures so that the child has good breastfeeding*. *I did not know these procedures—now I know*. *My advice is now better because of increased knowledge*.

–Community-based provider, Mogovolas

Many facility- and community-based providers also described how the job aids changed their counseling technique. While the original intent of the materials was to aid providers in their day-to-day work of identifying and counseling on breastfeeding problems, in practice, the majority of facility- and community-based showed the materials to the women they counseled. The images in the job aids aided providers in their explanation of proper breastfeeding positioning and latching correctly, and improved mothers’ comprehension. Most providers described asking mothers to breastfeed, observing and assessing their technique, and then adjusting the mothers’ positioning and latch to mirror the images in the job aid. In contrast to Phase 1 findings, where providers rarely observed or provided practical help to mothers, providers in Phase 3 described using the job aids to facilitate providing practical support (Table 3). Some facility- and community-based providers reported that the job aid images were particularly useful among community-based providers and mothers with low literacy, as illustrated in the quote below.

*It was easy to transmit information to breastfeeding mothers*, *because the job aid is made of images*, *so most of the people have low educational level*, *perceive more seeing figures*, *it is easier to explain how this breastfeeding is done*.

–Facility-based provider, Mogovolas

Providers also reported they provided more individualized counseling tailored to each mother’s circumstances and were able to establish a dialogue with the mother using the job aid (Table 3).

The job aids also facilitated incorporation of new topics into current breastfeeding counseling, including breastfeeding technique and problems. Post-rollout of the job aids, nearly all facility- and community-based providers used the job aids to counsel on breastfeeding techniques, such as signs of good latch and optimal positioning of the mother and infant (Table 3). All mothers reported receipt of counseling on technique and were able to describe and demonstrate proper positioning. Nearly all mothers appreciated learning about the “*rules*” for breastfeeding that they did not know before counseling, which included holding the breast with an open hand in the shape of a “C”, obtaining optimal positioning with the infant facing the mother’s chest, and ensuring proper latch.

*I was advised that I should not pick up the breast like this (showed the shape of scissors); I have to take this position (showed the hand in the shape of a “C”) for the child to suck well*. *I was also advised to hold my hand on the child’s bottom and control her feet*. *It helped a lot because I know this will help my daughter to suckle well*, *to gain weight and also it will help my own health*.

–Mother, Meconta

It is notable that both facility- and community-based providers relayed the job aids helped to resolve breastfeeding problems. Breast engorgement was the most commonly mentioned physical breast problem in facility- and community-based provider interviews in Phase 3. Preventing and managing mastitis was also mentioned, though more frequently among facility-based providers compared to community-based providers who provide referrals. As recommended in job aids, both types of providers reported advising mothers experiencing breast engorgement to continue breastfeeding and to empty each breast to reduce swelling and prevent infection.

*If we see that she has swelling*, *the child needs to breastfeed instead of stopping breastfeeding in that breast that has swelling*. *Then we must give the same breast constantly*, *to empty that milk because the thing that causes it to fill or to have this swelling is milk*, *is the fat that covers all the valves through which the milk comes out*.

–Community-based provider, Mogovolas

Mothers reported being advised to manage engorgement by continuing to breastfeed, expressing breastmilk, applying warm cloths to the breasts, or taking a warm shower to help express the breastmilk, strategies which were all described in the job aids.

*Yes [counseling] helped because I managed to express the milk out of the breast that always created a fever*, *now [I am] breastfeeding my son without pain or fever*.

–Mother, Mogovolas

The job aids also enabled increased provider awareness of breastfeeding problems or problems they had difficulty diagnosing. One facility-based provider appreciated the signs of mastitis listed in the job aid, as she relayed below:

*The job aid helps a lot*. *I use it a lot to help because not all the questions I have here in my head*. *For example*, *a mother comes in with complaints that she usually has fevers in the night*. *I talk*, *I am looking in my job aid and I find information that normally all the mothers with this complaint have a fever*.

–Facility-based provider, Mogovolas

While perceptions of breastmilk insufficiency emerged as a common breastfeeding problem, facility- and community-based providers were more likely to mention insufficient breastmilk compared to mothers. The job aids were used to demonstrate proper positioning and how to massage the breast to enhance breastmilk supply by facility and community-based providers.

*[Mothers] have complained that the [breast] milk does not come out*, *that the baby cries a lot and it does not satiate them*. *I ask them to take the breast and give it to their baby*, *but they continue to hold it not the best way*, *the scissors [hand formed in the shape of scissors when holding the breast]*. *After teaching what I learned in the poster*, *in my presence she finds the correct position and thanks me*: *“Thank you*, *I did not know*.*”*

–Facility-based provider, Mogovolas

Additionally, some providers reported that prior to the job aids, they primarily advised mothers to resolve insufficient breastmilk by changing their diet. However, the job aids provided them with additional management strategies that they found to be fruitful (Table 3).

Cracked nipples were also mentioned by providers, though community-based providers were more likely to report counseling on this problem than facility-based providers. Community-based providers reported that the job aid taught them about the causes of cracked nipples and helped them resolve this problem. Although community-based providers were likely to refer mothers with cracked nipples to the health facility, the job aid reinforced consistent counseling messages between the community and facility (see Table 3).

While the original intent of the job aids was to assist health providers in identifying and resolving breastfeeding problems, both facility- and community-based providers reported that the job aids also facilitated the prevention of various breastfeeding problems. The job aids drew new attention to counseling on breastfeeding, even in the absence of breastfeeding problems or child growth issues. Some facility-based providers described how the job aid encouraged them to counsel proactively on problems before they occurred so that mothers were prepared to solve these problems and continue breastfeeding.

*[The job aid] changed [counseling] because before*, *there was no counseling*. *The counseling that was available was if an infant was malnourished*, *I would look at the card and pay attention*, *say that you have to breastfeed*, *ask if there was another child at home*. *Before I only noticed the issue of breastfeeding*. *I had no concept before*.

–Facility-based provider, Mogovolas

Similarly, even among mothers who reported that they had not experienced any breastfeeding problems, many reported being counseled on the prevention of potential problems such as engorgement.

*I was also advised that there are times when the breast gets full of [breast]milk*, *I have to warm water to get a cloth to wet and to massage on and under [demonstration] because if it is a problem that the milk gets thick inside it will help to dilute the [breast]milk [to make the breastmilk more fluid]*.

–Mother, Meconta

#### Effect on provider self-efficacy and motivation

An increase in reported provider knowledge was accompanied by improved provider confidence and counseling self-efficacy. Community-based providers reported that showing mothers the job aid increased their credibility and gave them confidence that what they were saying was evidence-based. They relayed that mothers were more likely to listen to what they said because their words were *taken as fact* instead of based on experience or personal opinion. Both facility- and community-based providers reported that after implementation of the job aid, mothers were more likely to follow their recommendations, which improved their self-efficacy and motivation as they saw the effect the counseling had on their community. Providers were also motivated by seeing the improved child growth (i.e., weight gain) in their communities, which some attributed to their improved counseling skills and knowledge (Table 3).

*I get positive responses from the mothers themselves*. *Because whoever gives a lecture hopes to give information and the person will follow that information and put it into practice*. *I have received this from them [mothers]; I have had positive responses*.*… I felt more firm in my words*, *in what I said*, *what I pass on to the women—I have a certainty*.

–Facility-based provider, Mogovolas

Multiple providers reported that mothers returned to see them and thank them for their breastfeeding support.

*Yes*, *a mother after birth had sores on the nipple*. *After I counseled*, *the wounds healed and she became more confident that the materials helped her because two to three days [later] the mother again thanked me for the advice*. *At first*, *they did not come because they did not know that I knew how to help mothers with these problems*, *but after knowing they already came by themselves*.

–Community-based provider, Mogovolas

For some community-based providers, the increased knowledge and self-efficacy they gained from the job aid enabled them to solve more breastfeeding problems themselves and make fewer referrals to the health facility. A few community-based providers described having no context for counseling on breastfeeding problems and no training on how to manage them prior to being trained on the job aid. The job aid allowed them to attempt to solve breastfeeding problems before making a referral (Table 3).

#### Improvements to the job aids

The majority of providers described that the job aid format and content was helpful and easy to use for counseling on breastfeeding problems. Minor suggestions to improve the job aid content and format are shown in Table 4.

**Table 4:** Job aid improvements proposed by facility- and community-based providers, Phase 3 findings.

Proposed modifications to the job aids	
**Content modifications**	Clarify breastfeeding recommendations for HIV-positive women and orphan children Simplify vocabulary for low literacy community-based providers and mothers Translate job aids into local language of Macua
**Format modifications**	Enlarge images to show mothers Provide poster size for health facility walls and pocket size for home visits Reformat into booklet format with page numbers

## Discussion

This implementation science study revealed that breastfeeding challenges, such as inadequate latching, poor positioning, perceptions of breastmilk insufficiency and breast engorgement, were common barriers to EBF. Maternal perception of insufficient breastmilk was the most frequently reported challenge to sustaining EBF, according to mothers, facility and community-based health providers. Recent evidence corroborates these findings on perceived insufficient breastmilk [8,19–22], which has been associated with breastfeeding cessation and early introduction of foods and liquids prior to the recommended six months of age [23–27]. In addition, these findings point to inadequate maternal dietary intake as the primary reported cause of decreased breastmilk quantity. A qualitative assessment in four provinces of Mozambique reported that maternal consumption of certain foods, such as peanuts and coconut, were advised as a way to resolve breastmilk inadequacies [11]. Diminished breastmilk quantity and quality reported in Democratic Republic of Congo, Eastern Uganda, Egypt and Kenya, showed that “*eating well*”, “*strength and energy giving foods*” and “*sufficient amounts*” were related to mothers’ adequacy of breastmilk production [22,28–31]. Yet, maintaining breastfeeding frequency and duration (i.e. emptying both breasts), as the recommended way to increase breastmilk production was rarely mentioned.

The lack of knowledge and skills to address the common perception of insufficient milk led health providers to refer mothers to the INAS program to receive infant formula. This was compounded by lack of time to provide counseling and the lack of knowledge on breastfeeding physiology. The Multisectoral Action Plan for the Reduction of Chronic Undernutrition in Mozambique 2011–2020 (PAMRDC) and the national IYCF strategies call for training of community health cadres (i.e., volunteers and community health workers) and community peer support groups on breastfeeding physiology, and coordination between the health and social action sectors, as possible solutions. While the MOH has an action plan to disseminate the Code for Marketing Breastmilk Substitutes and forge links with the National Inspection for Economic Activities to monitor the Code’s implementation, our findings support a need to strengthen coordination between the health and the social action sectors. Guidance on referrals to social protection programs, adherence to program eligibility criteria to ensure appropriate and safe distribution of infant formula, dissemination of information on risks of infant formula, and monitoring of free or subsidized programs for breast-milk substitutes are needed to ensure proper use of infant formula in special contexts.

In Mozambique, latching problems and breast engorgement emerged as key challenges to EBF described by health providers. This likely reflected the lack of pre-service training and the inadequate capacity of lay health professionals to provide practical lactation support for mothers at routine contact points. Several studies have shown that women experiencing physical breast problems were more likely to cease EBF within the first six months of life in Democratic Republic of Congo and at 4, 12, or 22 weeks after delivery in Nepal [32,33]. In Mozambique, while community-based providers reported receipt of some in-service training for breastfeeding counseling, lack of self-efficacy led to health facility referrals for care of breastfeeding problems. Facility-based providers did not receive in-service training for management of common breastfeeding problems or breastfeeding counseling skills in Phase 1, prior to rollout of job aids.

Following the rollout of the job aids, Phase 3 findings reveal that the job aids re-affirmed existing health provider knowledge, and both facility- and community-based providers shared that they learned new information about breastfeeding problems and technique. Post-rollout of the job aids, providers delivered more interactive counseling, and actively assessed breastfeeding techniques, alongside provision of practical support, which was valued by mothers. This translated into a greater number of breastfeeding challenges identified through the job aids, earlier resolution of breastfeeding problems before they became severe, as well as prevention of problems, in some cases. The job aids were also effective in improving provider confidence and self-efficacy in counseling. Community-based providers were better equipped to solve a greater number of breastfeeding problems, with fewer referrals to the health facility. Regular post-training supervision during implementation was fundamental to the reinforcement of counseling skills and use of the job aids. While it was not the original intent of the job aids, the results indicate that the use of the job aids also had a positive effect in improving providers’ motivation, as many providers referred to being happy that mothers were returning to thank them for the support.

Similarly, other studies have described the use of job aids during antenatal and postnatal provider counseling contributing to improvements in counseling quality and maternal knowledge [34–36]. A pre-post randomized study on the effectiveness of a job aid in improving postnatal counseling among facility-based providers in Benin found significantly increased frequency of provider counseling on targeted topic areas in newborn health, including EBF [34,35]. Following a three-day provider training and continued field support throughout the intervention period, the intervention group showed significantly higher maternal knowledge compared to baseline, which demonstrated that intensified provider counseling led to improved maternal understanding and retention of information. In Tanzania, a quasi-experimental study evaluated an integrated program to address provision of infant feeding counseling to HIV-positive mothers. The intervention consisted of health provider job aids, take-home materials for mothers, and provider training on effective use of the job aids and counseling skills [36]. Mothers in the intervention group reported receiving higher quality counseling and showed increased maternal knowledge of EBF recommendations compared to the control group.

The effectiveness of practical support in addressing breastfeeding challenges and improving rates of EBF is also substantiated in the literature. In a community-based intervention in Bangladesh, community health workers received a 21-day training, which included modules on breastfeeding counseling, practical observation, assessment of technique, and common breastfeeding problems. The trained health workers identified poor breastfeeding positioning and attachment and provided practical support to help mothers establish breastfeeding during home visits [37]. Mothers in the intervention arm had better success overcoming breastfeeding difficulties compared to those in the control group. In a qualitative study of peer-based support for EBF in Uganda, the provision of individualized counseling and practical support by peer counselors was well received by mothers, who appreciated receiving information relevant to their particular situation and assistance with latch and positioning [38].

While this study showed that job aids can address the variation in capacity between health facility- and community-cadres to address breastfeeding challenges and be tailored to specific contact points, quality trainings and supportive supervision are key to building skilled lactation support at the country level. Based on study findings, pre- and in-service trainings can include topics such as breastmilk physiology and breastmilk production, especially milk let down, infant nutritional needs and stomach capacity in the initial days postpartum; practical skills on breastfeeding techniques; and how to identify and resolve physical breast problems (e.g., manual expression techniques to alleviate engorgement, cracked nipples and wounds). Providers can also be equipped to provide anticipatory guidance to mothers after birth [12], including what to expect when milk transitions from colostrum to mature milk and engorgement is more likely; what tips to give when women return to work on the farm; or that cracked nipples can actually be a sign of a bad latch. The provision of anticipatory guidance can be integrated into on-the-job training and supportive supervision, which was notably weak.

While building the capacityof health providers by equipping them with job aids to improve the provision of quality breastfeeding counselling, identified system-level issues were not addressed in this study, including lack of sufficient human resources and adequate infrastructure to provide privacy during breastfeeding counseling and support sessions in health facilities. This needs to be addressed through advocacy efforts for adequate planning, resource allocation, and use of data and evidence for decision making.

### Limitations

This study had a few limitations. A purposive sample was used which could lead to a low external validity of these findings. However, it is important to note that we systematically defined a priori the inclusion and exclusion criteria to identify potential study participants and recruitment involved selection of a diverse sample with regards to socio-demographic characteristics of women and health providers. While we acknowledge that the findings of this study cannot be generalized to the entire population, this implementation science study can provide greater understanding on how and if job aids can be useful to help health providers improve counseling on key breastfeeding challenges, taking into account diverse socio-economic and cultural factors. All but three breastfeeding counseling sessions were conducted per the suggestion of the research team to observe breastfeeding counseling. Despite this limitation, health professionals were unable to demonstrate an adequate level of understanding of breastfeeding counseling and lactation support. During Phase 1 counseling observations, though it was the intention to interview only mothers who had experienced breastfeeding problems, mothers were approached about participating in IDIs prior to their appointments at the health facility, making it difficult to identify mothers with breastfeeding challenges. This resulted in a relatively small sample of mothers who had experienced breastfeeding problems and could speak to their experiences managing problems. Key challenges experienced during the course of the study, which were not addressed in the job aid, included language barriers for providers and clarification on when to appropriately use infant formula for special circumstances (i.e. vulnerable infants, including orphans and HIV-affected infants). In addition, the duration of job aids for three months occurred during the last quarter of MCSP project implementation in Nampula province. Yet, the success of the job aids was facilitated by the support of two nutrition officers based at the district-level in both study districts, with one officer focused on health facility-level support and the other focused on community-level support. This allowed for frequent mentoring of health providers that participated in Phase 3. While it is possible that these results may not be sustained in the absence of such support, or availability of job aids, providers had the knowledge needed to use the job aids, and were motivated with the positive results, which may contribute to sustained use of the job aids. The roll out period may have been too short and it is not known whether facility and/or community based health providers continued to use the job aids following the end of MCSP project, especially without the support of frequent mentoring. Monitoring EBF rates in those locations, disaggregated by health area, would likely aid in demonstrating to health providers the need to use the materials and provide quality counselling. Such monitoring is currently possible with the new child health registers that have recently been rolled out in country.

In Phase 3, MCSP nutritionists at the facility level selected health facility providers to participate in IDIs, which may have introduced selection bias, as identified providers may have used the job aid frequently, and/or liked the job aid. Observations of breastfeeding counseling sessions were not repeated after the job aids were distributed, so findings relied entirely on upon self-report which should be taken into account when interpreting these findings. Finally, during Phase 3, health providers working in maternity wards in the selected study sites were not available to participate in the follow-up interviews, which limited assessment of the usefulness of the job aid in maternities.

While this study did not target women during pregnancy, which is a limitation, it is essential to prepare mothers to breastfeed during antenatal care [39]. Consideration should be given to how job aids can be adapted for the antenatal context and how breastfeeding counseling during antenatal care can be strengthened. There is growing evidence that provision of breastfeeding counseling during pregnancy can prepare women to resolve early breastfeeding challenges and improve EBF duration. In a systematic review conducted by the WHO, postnatal breastfeeding counseling as a singular intervention demonstrated 11% lower likelihood of *not exclusively breastfeeding* at 6 months in comparison to both antenatal and postnatal counseling, which showed a 29% reduction in not EBF (compared to standard of care or no breastfeeding counseling) [12]. A randomized controlled trial on the effect of breastfeeding counseling on EBF rates in Ghana found that women receiving breastfeeding-specific counseling in the (1) ante-, peri- and postnatal periods and (2) just the peri- and postnatal periods were significantly more likely to exclusively breastfeed for 6 months compared to women receiving only general health counseling during these times [40]. Other randomized trials in Bangladesh and Mexico testing breastfeeding-specific counseling interventions in the antenatal and early postpartum periods yielded similar improvements in EBF duration [41–43]. In addition, while this study provides valuable qualitative information on type and quality of breastfeeding counseling within the context of project implementation, a pre-/post- intervention study design, with a longer duration of intervention rollout, alongside additional systematic interviews and observation of breastfeeding counseling amongst different types of health workers and mothers may be warranted.

### Key Recommendations

**Fig 3:**
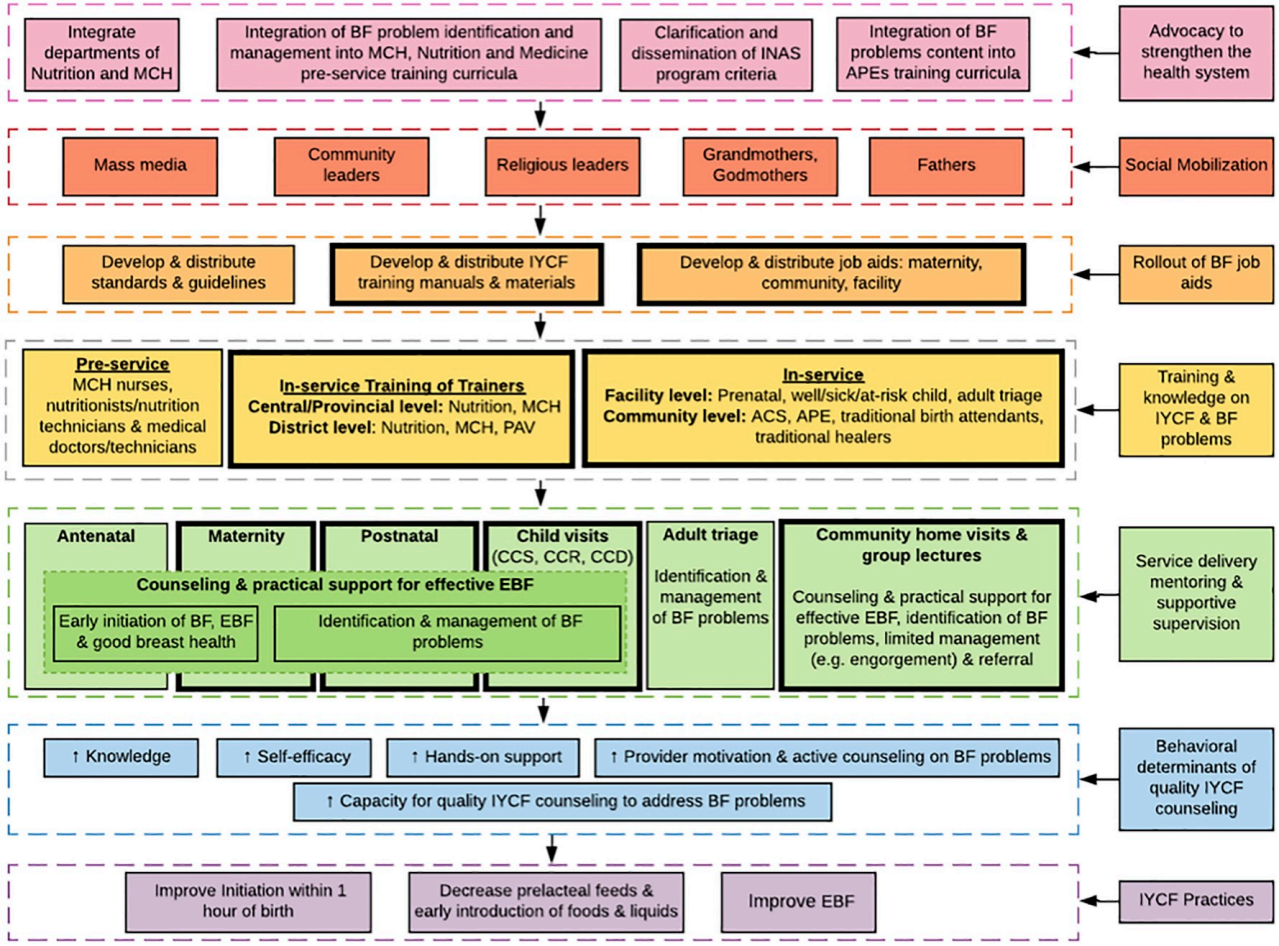
Program impact pathways analysis: Addressing breastfeeding challenges in Nampula, Mozambique. This is Fig 3 legend: Bold-bordered boxes: MCSP intervention area under the scope of this study. ACS: Activista comunitário de saúde (community health activists, community health workers generally associated with community-based or non-governmental organizations); APE: Agente Polivalente Elementar (Government Community Health Worker); BF: breastfeeding; CCD*: sick child visit; CCR*: at-risk child visit; CCS*: well child visit; EBF: exclusive breastfeeding; INAS: Instituto Nacional de Acção Social (National Institute of Social Action); IYCF: infant and young child feeding; MCH: maternal and child health; PAV: Programa Alargado de Vacinação (Expanded Program in Immunization). *Portuguese.

Building the capacity and skills of health providers at facility and community levels in lactation management, especially at childbirth and the first days following birth [44], combined with strong monitoring of formula distribution, provides a unique opportunity to strengthen EBF counseling in this area of Mozambique (see Table 5). The MOH adapted UNICEF’s community-based IYCF counseling package, which includes IYCF counseling cards and a key message booklet. While these materials include illustrations and messages appropriate breastfeeding techniques, such as positioning and attachment, and address barriers such as mother and infant separation at childbirth, they do not include step-by-step guidance for providers on how to respond to challenges to EBF presented by mothers. This study revealed that the MOH IYCF counseling package has been used primarily for breastfeeding promotion activities, and the job aids developed during this study would complement the MOH IYCF package. Husbands were mentioned by mothers and providers as the primary source of support for EBF, which is consistent with the Strategy for Social and Behavior Change Communication for the Prevention of Malnutrition in Mozambique [15] and indicates the importance of breastfeeding counseling reaching influential family members. Moreover, several key actions can be undertaken to improve EBF counseling in the Mozambique health system, which are categorized into advocacy, social mobilization and behavior change communication interventions (Fig 3).

- **Advocacy interventions** can be implemented to ensure the integration of content on the identification and resolution of breastfeeding problems and challenges in relevant training curriculums, programs, and departmental objectives across the MOH and other sectors, such as Gender, Child and Social Action Sector and the National Institute of Social Action (Instituto Nacional de Acção Social [INAS]) to ensure that government programs are harmonized.
- **Social mobilization interventions** can involve mass media, community leaders and key family influencers to address EBF challenges.
- **Behavior change communication interventions** include the rollout of breastfeeding job aids and development and distribution of associated materials to facility- and community-level; equipping health workers with the knowledge and skills needed to counsel on IYCF and breastfeeding challenges; training of health providers, service delivery, mentoring and supportive supervision to ensure the quality of counseling and support in identified contact points.

**Table 5:** Key programmatic recommendations, based on study findings.

Recommendation	Rationale
Update existing maternal and child health and nutrition guidelines and standards	Feature breastfeeding challenges and problems in key guidelines (i.e., national antenatal care guidelines), standards, and supportive supervision tools for facility-based health providers to improve breastfeeding counseling during antenatal care, maternity/childbirth, postnatal and child health services.
Update pre-service curricula	Integrate breastfeeding counseling content in pre-service curricula for facility and community-based health providers and develop supportive supervision tools for community-based provision of nutrition services, including breastfeeding counseling [12,45] (e.g., as part of the Global Financing Facility Disbursement-Linked Indicator 4, Package of Nutrition Interventions)
Provide in-service training and supportive supervision for health providers and integrate communication skills into on-the-job training	Emphasize support for early breastfeeding initiation (e.g. early breastfeeding physiology, colostrum, breastfeeding techniques) and management of common breastfeeding problems (e.g. sore nipples, breast engorgement and mastitis, breastfeeding challenges faced by working women, latching and insufficient milk). Incorporate listening and learning skills, build confidence and self-efficacy, train providers to give anticipatory guidance, and include provider behavior change to address cultural beliefs and attitudes on breastfeeding challenges into on-the-job training.
Complement existing IYCF materials with job aids	Validate and roll out job aids in complement to Mozambique’s MOH adapted UNICEF C-IYCF Counseling Package cards at a subnational and national scale. These tools that can help strengthen the quality of breastfeeding counseling in both community and facility settings [6,45].
Provide practical support to mothers through health providers, who can identify and manage breastfeeding problems and prevent future problems from occurring.	Help mothers prevent problems by addressing the benefits of adopting good practices, analyzing the cause of any breastfeeding difficulty or problem and suggest ways to help resolve the difficulties.
Support skills in breastfeeding observations	Equip health providers with skills to observe the interaction between mother and baby during routine consultations, be able to answer the mother’s doubts about breastfeeding and care of the infant, and aid in supporting the baby’s latch and positioning. A standard breastfeeding history form as part of the patient records for the visit may help institutionalize the practice of observing and assessing breastfeeding technique.
Update job aids	Address literacy and language barriers faced by the providers in the design of breastfeeding counseling trainings and associated materials, particularly at the community level. Address confusion and concerns for feeding recommendations for orphans, vulnerable children, children exposed to HIV, and women who believe their breastmilk is insufficient. Clarify the use of infant formula—for whom and when.
Integrate the job aids with the Baby Friendly Hospital Initiative	While the government of Mozambique has prioritized breastfeeding, BFHI has waned in Mozambique. No health facilities or hospitals are certified as baby friendly in Mozambique since its inception in 1998–1999, and revitalization in 2007, and rollout of trainings in central and provincial hospitals in 2010–2011. While these hospitals have ongoing implementation of BFHI, support is needed to achieve certification. Promote early breastfeeding initiation and counseling on EBF in maternity wards through strengthening implementation of BFHI to include Kangaroo care and respectful maternity care, updating local BFHI guidelines (Mozambique currently uses Brazil’s BFHI guidelines [46]). Update training, behavior change and supportive supervision materials in line with recent WHO BFHI guidelines, alongside job aids for addressing breastfeeding problems [17].
Task shift to community-level health workers for comprehensive breastfeeding support.	Health providers relayed that excessive caseloads, lack of time available to counsel mothers, and lack of privacy to perform one-on-one counseling at the health facility level. Task shift to community-based health workers for comprehensive breastfeeding support, as a key strategy for improving EBF outlined in the PAMRDC and the IYCF Strategies, should be considered. There are several breastfeeding support groups led by community providers or mother-to-mother groups, overseen by health units, which provide individual and group counseling and support. The lead health units could provide the necessary support and materials, as well as strengthened communication between health services and the community level.
Address early return to work by creating supportive work spaces, social networks and communities.	return to work postpartum was reported as a key challenge to EBF in Mozambique, and has been described in the literature [47,48]. Mozambique labor law is currently under review to increase maternity leave from 60 days to 90 days and paternity leave from one day to seven days. Extension of maternity leave to 18 weeks [49], as recommended by the International Labor Organization, and establishing protection measures for women in the informal sector (i.e., enforcement of maternity leave, daycare centers) is an important consideration for revisions of the labor law [47,50]. Create baby friendly work spaces and build a supportive social network through community day care centers in rural areas, such as Nampula, where many mothers work as farmers. Family members (i.e., grandmother, aunt, older sibling) can also benefit from understanding how they can support breastfeeding at the household level, and how to feed the baby appropriately in the mother’s absence. This may reduce the burden placed on older siblings in providing care for infants and prevent absenteeism from school in the long term.

As a result of the implementation of these interventions, it is expected that behavioral determinants of quality IYCF counseling will be addressed and improved, including knowledge, self-efficacy, practical support, and provider motivation and capacity; and this in turn will improve IYCF practices, including increased EBF, as described in our Program Impact Pathways, an adaptation of Nguyen et al, 2014 [14] Analysis, Fig 2.

Key recommendations based on the findings of this study are described in Table 5.

## Conclusion

In the future, findings from this seminal implementation science study on breastfeeding counseling can be used to integrate high-quality breastfeeding counseling content into maternal and child health and nutrition curricula and supportive supervision materials for community and facility levels that address IYCF. The *2018 WHO Guidelines on Counseling of Women to Improve Breastfeeding Practices* recommend counseling occur during at least six breastfeeding contacts, which includes antenatal care, the first two to three days of life, the neonatal period (first week), and early infancy (first three to four months), which can be leveraged in Mozambique to address challenges to EBF at key time points and with appropriate frequency [12]. Furthermore, the development of clear lactation management protocols that help providers understand the causes and key ways to address insufficient breastmilk, physical breast problems (engorgement, sore nipples), positioning, and correct latching would be of use in Mozambique. Evidence indicates that breastfeeding problems tend to originate from poor lactation knowledge and management very early on after birth, so it is important to reach women with effective breastfeeding counseling support in health care facilities and at the community [44]. Our findings also call for addressing the excessive caseloads and limited time that health providers have through task shifting to community health workers, as well as early return to work postpartum which is a key challenge to EBF in Mozambique. Finally, as Mozambique has endorsed the WHO Code for Marketing Breastmilk Substitutes [51,52], the MOH and Ministry of Gender, Child and Social Action should join efforts to ensure the criteria for infant formula distribution to INAS are known by all relevant stakeholders and strictly followed.

Short-term and long-term investments to improve breastfeeding counseling services during routine contact points and the implementation and sustainability of large-scale improvements in breastfeeding counseling in Mozambique would benefit from employing the Breastfeeding Gear Model as a framework. The Breastfeeding Gear Model can provide countries, such as Mozambique, with practical guidance to address gaps in breastfeeding counseling and propose policy recommendations to strengthen the breastfeeding environment across a number of domains [53,54]. Specifically, the Breastfeeding Gear Model examines what is needed to scale up EBF counseling through eight gears: advocacy, political will, legislation, funding and resources, training and program delivery, promotion, research and evaluation, and coordination and monitoring. Collectively, harmonization of these components is needed to achieve improvements in the scale-up of breastfeeding-friendly environment and in building country capacity in skilled lactation care, which is essential to improve breastfeeding practices globally [55].

## Supporting information

S1 Fig Job aid—Job aid for maternity ward/childbirth. (PDF)

S2 Fig Job aid for child health/ postpartum health services. (PDF)

S3 Fig Job aid for community health workers. (PDF)
