# Supplementary material for: Strengthening counseling on barriers to exclusive breastfeeding through use of job aids in Nampula, Mozambique
Source: PLoS One. 2019 Dec 2;14(12):e0224939. doi: 10.1371/journal.pone.0224939 (PMC6886792; doi:10.1371/journal.pone.0224939)
Supplement: S1 Fig — (PDF) [file pone.0224939.s001.pdf]

REPÚBLICA DE MOÇAMBIQUE
Ministério da Saúde

MATERIAL DE APOIO PARA PESSOAL DE SAÚDE QUE APOIA O ALEITAMENTO MATERNO NA MATERNIDADE E PARTEIRA TRADICIONAL

1. Apoie a mãe a colocar o seu bebé pele-a-pele logo após o parto e por pelo menos uma hora.

- O contacto pele-a-pele ajuda a acalmar a mãe e o bebé e ajuda a estabilizar o batimento cardíaco e a respiração do bebé.
- O contacto pele-a-pele promove o início da amamentação na primeira hora.

Explique a mãe:

- O bebé deve ficar junto a si para ele ficar quentinho e encontrar a mama facilmente.
- Iniciar cedo a amamentação ajuda o bebé a aprender a mamar bem.
- Iniciar cedo a amamentação ajuda a reduzir o seu sangramento após o parto.

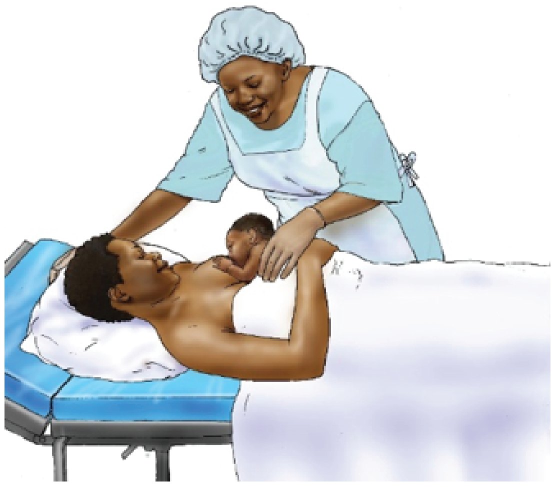

2. Incentive a mãe a dar o colostro ao seu bebé.

- O colostro é muito rico em nutrientes e tem água para o bebé.
- O colostro transfere imunidade da mãe para o bebé, protegendo-o de doenças.
- O colostro ajuda a passar o mecónio, as primeiras fezes escuras e pegajosas.

Explique a mãe:

- Nestes primeiros dias vai sair um leite grosso, que pode ser um pouco amarelo ou de cor clara, que se chama colostro.
- O colostro tem muitas vitaminas e é bom para o seu bebé.
- O colostro é como a primeira vacina do bebé, que lhe protege de todas as doenças que a mãe já teve.
- O colostro ajuda a tirar as primeiras fezes escuras e pegajosas, por isso o bebé não precisa de tomar medicamentos tradicionais.
- O colostro sai em pequena quantidade nos primeiros dias. Isto é suficiente para o seu bebé, e já tem água para matar a sua sede.

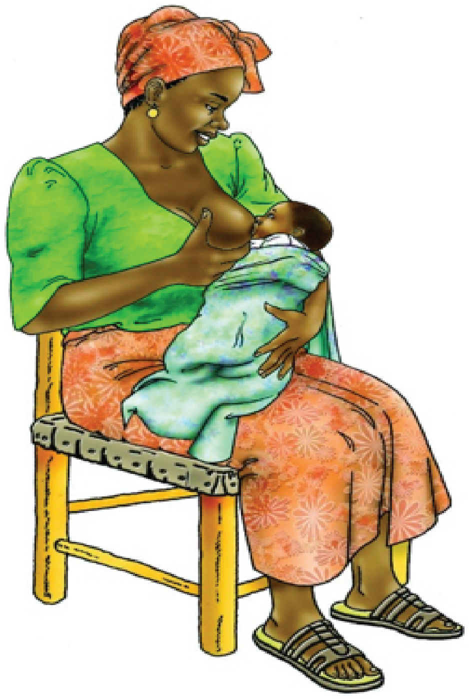

3. Não dê alimentos pré-lacteos e desencoraje a mãe de dá-los ao bebé. Se o bebé ainda não iniciou a alimentação: é importante não dar outros alimentos ou líquidos além do leite materno pois podem dificultar a amamentação e causar doenças.

Se o bebé iniciou alimentação pré-lactea mas a mãe está em condições de amamentar, explique a mãe:

- O seu bebé não precisa de outros alimentos ou líquidos, só leite materno.
- Dar água ou outros líquidos pode trazer doenças para o bebé.
- Coloque o bebé ao peito sempre que ele quiser, pelo menos a cada 2 ou 3 horas, de dia e de noite.

Sempre que necessário dê ajuda prática à mãe para iniciar a amamentação.

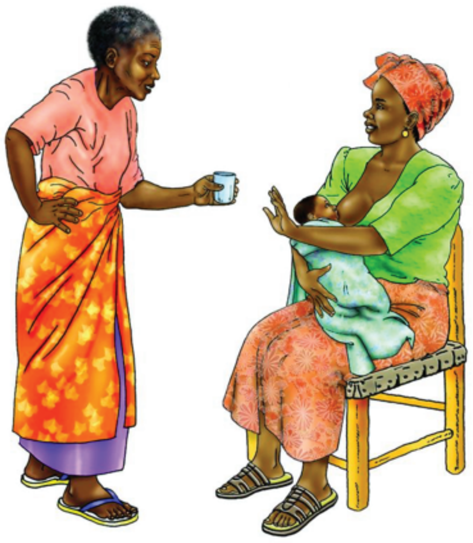

USAID DO POVO AMERICANO

Maternal and Child Survival Program
Programa de Saúde e Sobrevivência Materna e Infantil

4. Dê ajuda prática nas primeiras mamadas.

- Muitas vezes uma ajuda prática é melhor do que explicar muitas coisas, e mostra que o profissional está pronto para apoiar a mãe.
- Ajude a mãe principalmente se este é o seu primeiro bebé, se tem gémeos, ou se ela tem um problema prático evidente: está desconfortável, suja, cansada ou se já recebeu muitas informações.
- Ajude a mãe a reconhecer o temperamento do seu bebé e a aprender a melhor maneira de responder às necessidades dele.

Sinais de fome

- Aumenta os movimentos dos olhos fechados ou abertos.
- Abre a boca, estica a língua e vira a cabeça para procurar a mama.
- Faz sons suaves de gemido.
- Chupa ou morde as mãos, dedos, manta ou lençol, ou outro objeto que entra em contato com a boca.

Mantenha a mãe e o bebé juntos, dia e noite.

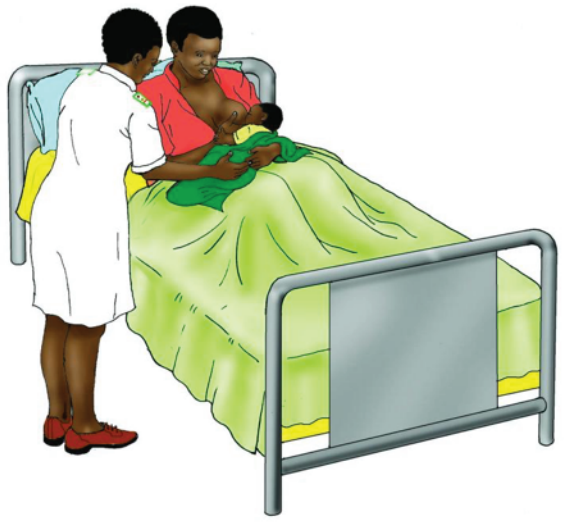

5. Ajude a garantir um bom posicionamento para a mamada ser confortável para a mãe e para o bebé.

Observe a mamada e verifique os seguintes aspectos:

- O corpo do bebé deve estar direito (cabeça, costas e rabinho alinhados).
- A cabeça pode estar ligeiramente esticada para trás.
- O corpo do bebé deve estar virado para o peito da mãe.
- O corpo do bebé deve estar encostado à barriga da mãe (barriga com barriga).
- Todo o corpo do bebé debe estar apoiado na mão e braço da mãe.
- A mãe deve estar confortavelmente sentada e com os pés apoiados.

Sempre que necessário dê ajuda prática.

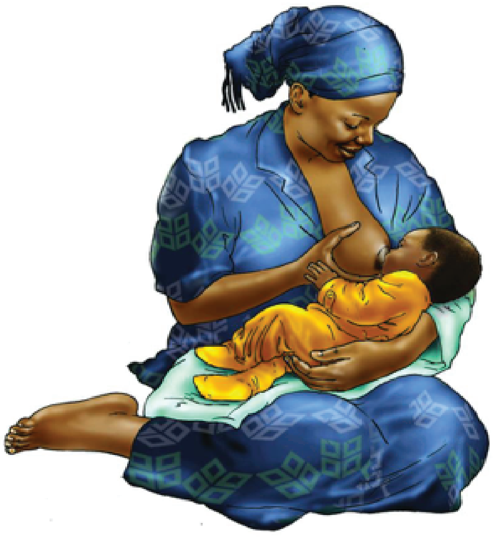

6. Ajude a garantir uma boa pega para evitar dores nos mamilos e para o bebé mamar bem.

Observe a mamada e verifique os seguintes aspectos:

- A boca do bebé deve estar bem aberta (pode estimular a abrir bem a boca exprimindo algumas gotas de leite materno para o bebé sentir o cheiro e tocando o lábio do bebé com o mamilo).
- O lábio de baixo do bebé deve estar virado para fora.
- O queixo do bebé deve tocar a mama da mãe.
- A mãe deve segurar a mama em formato de “C”, ter 4 dedos abaixo da mama e o dedo grande em cima a massagear a mama.
- Não se deve ouvir o barulho do bebé a chupar, mas pode se ouvir o bebé a engolir.
- As bochechas do bebé geralmente ficam cheias quando chupa.

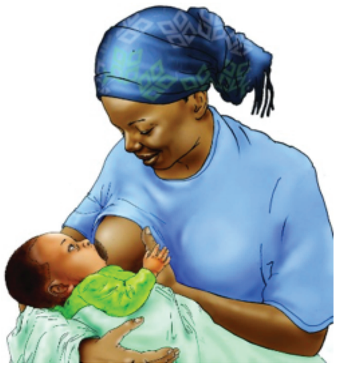

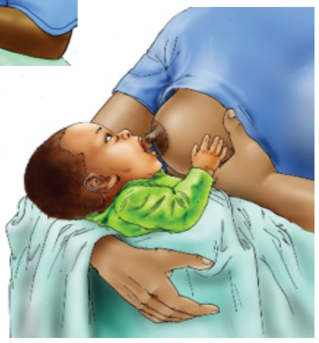

7. Observe as mamas da mãe e apoie se ela tiver mamilos planos ou invertidos.

Explique a mãe:

- Pôr o bebé a mamar ao peito vai ajudar a puxar os mamilos para fora.

Dê ajuda prática:

- Ajude a mãe a posicionar o seu bebé nas primeiras mamadas. É importante dar essa orientação cedo
- Ajude a mãe a fazer o mamilo ficar mais saliente rodando-o entre os dedos antes de uma mamada para tornar a pega do bebé mais fácil.
- Pode usar uma seringa para ajudar a puxar o mamilo para fora, se necessário.

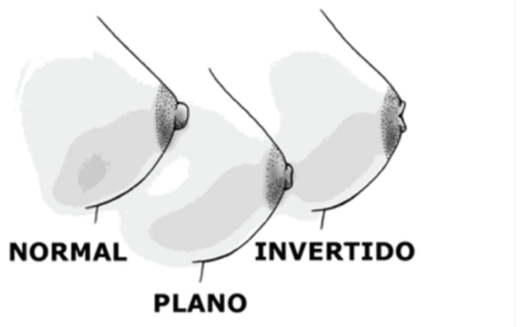

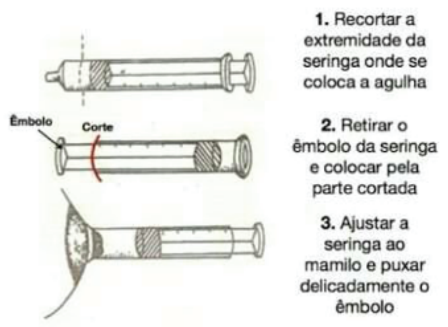

8. Aconselhe a mãe sobre como manter a saúde das mamas.

Explique a mãe:

- Quando o leite começar a descer, é possível que as suas mamas fiquem inchadas porque o bebé tem um estômago pequenino e não come muito a cada mamada.
- Quando as mamas incham por estarem cheias de leite pode sentir desconforto e, se não aliviar, isto pode gerar outros problemas mais sérios.
- É importante amamentar de ambas as mamas tantas vezes quanto o bebé quiser, de dia e de noite, e evitar intervalos longos entre as mamadas.
- Se as mamas incharem, ponha panos limpos e quentes nas mamas, ou tome um banho com água morna para ajudar o leite a sair. Depois, tire o leite manualmente (demonstre). Isto irá lhe aliviar.
- Se tiver dor nos mamilos ou nas mamas, se tiver feridas nos mamilos, se a pele das mamas mudar de cor, se sentir um caroço numa das mamas, se tiver febre ou qualquer outro problema com as mamas ou com a amamentação, vá à Unidade Sanitária logo que possível.

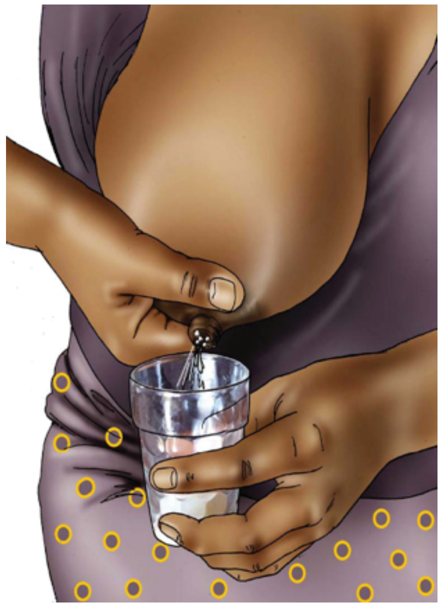

9. Aconselhe a mãe sobre como manter uma boa produção de leite.

Explique a mãe:

- Todas as mães são capazes de produzir leite suficiente para os seus bebés.
- Amamente muitas vezes durante o dia e a noite (a cada 2-3 horas ou sempre que o bebé quiser).
- Quando der de mamar, dê uma mama de cada vez até ficar leve e macia. Se o bebé quiser, ofereça a outra mama até ele largar sozinho.
- Aumente a quantidade de comida nas suas refeições e inclua 1-2 lanches entre as refeições.
- Beba muita água limpa e fervida ou tratada com certeza (pelo menos 8 copos por dia).
- Peça apoio da família para fazer as tarefas diárias e descanse o suficiente.

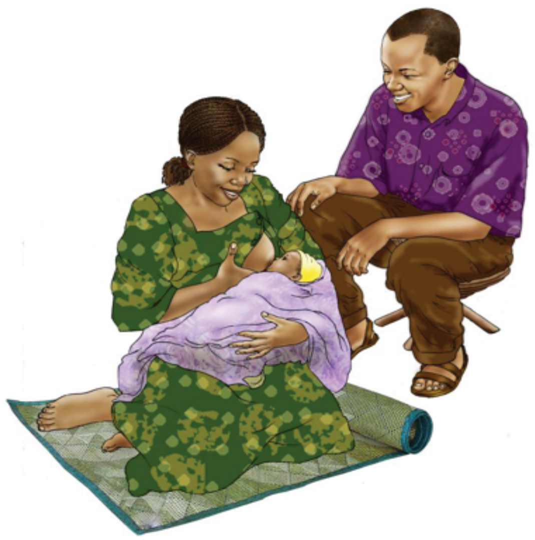

10. Apoie a mãe a amamentar em situações especiais.

Bebé com dificuldade para pegar a mama.

- Observe a mamada e identifique causa.
- Aconselhe e apoie a mãe no posicionamento e pega.
- Acalme o bebé e tente novamente, mas não force o bebé a mamar.
- Observe a mamada e identifique a causa.
- Incentive o contato pele-a-pele entre a mãe e o bebé em um ambiente calmo.
- Apoie na extracção do leite e ofereça com colher ou copo, se necessário.
- Tente novamente em meia hora.

Acordar um bebé sonolento que mama pouco.

- Retire as mantas e roupas pesadas para permitir a movimentação dos braços e pernas.
- Massageie com carinho o corpo do bebé e converse com ele.
- Evite cutucar ou aleijar o bebé com batidas na bochecha ou nos pés.
- Apoie a amamentar com o bebé numa posição mais vertical.
- Tente novamente em meia hora.

Acalmar um bebé que esteja a chorar.

- Os bebés podem chorar por fome, dor, fralda suja, solidão, cansaço e outras razões. Por isso, é importante oferecer apoio e desenvolver a confiança da mãe na sua capacidade de cuidar do seu bebé.
- Procure a causa do choro. Deixe o bebé confortável – fralda seca e limpa, mantas secas e não quentes demais.
- Coloque o bebé na mama. O bebé pode estar com fome ou sede e, às vezes, quer apenas chupar porque isso faz-lhe sentir-se seguro.
- Acaricie ou massageie gentilmente os braços, pernas e costas do bebé.
- Coloque o bebé no peito da mãe em contato pele-a-pele. O calor, o cheiro e o batimento cardíaco da mãe ajudarão a acalmar o bebé.
- Converse, cante e balance o bebé próximo a si.

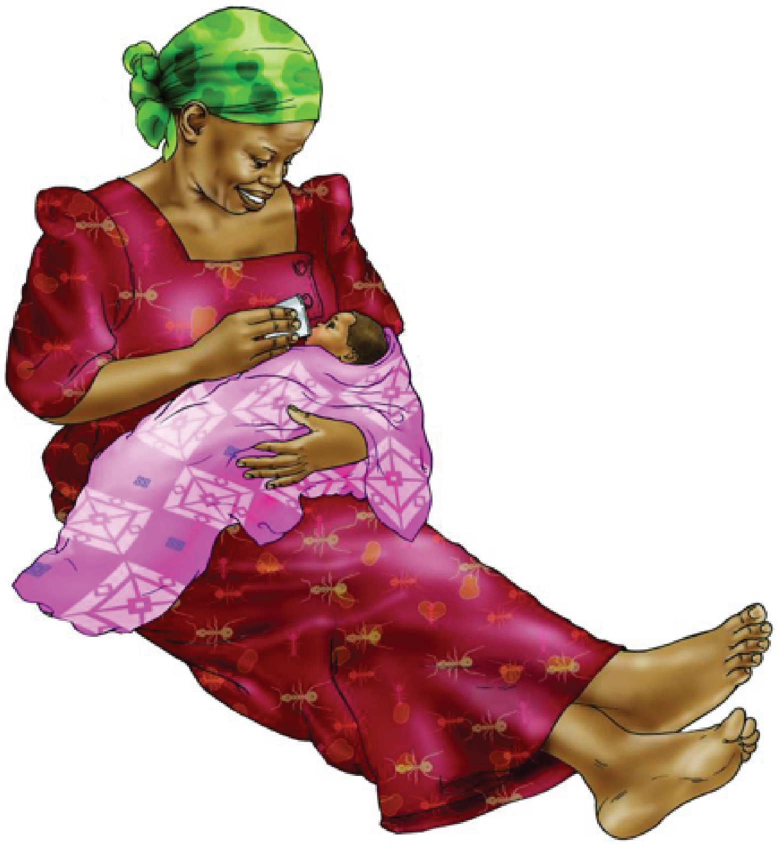

Técnicas para oferecer o leite materno ordenhado

Copo: para bebés com dificuldade para sugar mas capazes de engolir.

Colher: para bebés que não conseguem controlar o fluxo e têm risco de aspiração.

Seringa ou conta-gotas: para quantidades muito pequenas de leite, por exemplo, o colostro.

Sonda nasogástrica ou orogástrica: para bebés que não consegum sugar e engolir.

Retirada directa para a boca do bebé: apropriado para encorajar o bebé a sugar e para bebés com fenda palatina.
