# Supplementary material for: Strengthening counseling on barriers to exclusive breastfeeding through use of job aids in Nampula, Mozambique
Source: PLoS One. 2019 Dec 2;14(12):e0224939. doi: 10.1371/journal.pone.0224939 (PMC6886792; doi:10.1371/journal.pone.0224939)
Supplement: S2 Fig — (PDF) [file pone.0224939.s002.pdf]

REPÚBLICA DE MOÇAMBIQUE
Ministério da Saúde

MATERIAL DE APOIO PARA PROFISSIONAIS DE SAÚDE QUE APOIAM O ALEITAMENTO MATERNO (PARTE 1)

1 **Pergunte:** Como está a correr a amamentação?

BEM → 2 | **MAL** → 2

2 **Peça:** Posso ver como é que o bebé mama?

3 Verifique: BOM POSICIONAMENTO

- O corpo do bebé está direito e virado para o peito da mãe.
- O corpo do bebé está encostado à barriga da mãe (barriga com barriga).
- Todo corpo do bebé está apoiado na mão e braço da mãe.
- A mãe está confortável e não refere dor.

SIM

Elogie à mãe e passe para o ponto 4

Muito bem! É assim mesmo que se põe o bebé a mamar.

NÃO

Verifique a boa pega e passe para o ponto 4

Ajude a mãe a posicionar bem o bebé.

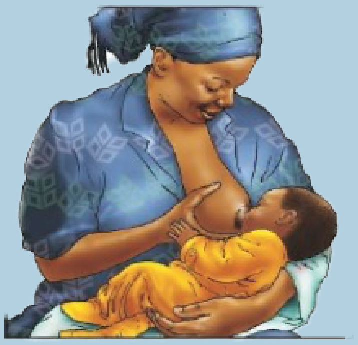

4 Verifique: BOA PEGA

- A boca do bebé está bem aberta.
- O lábio de baixo do bebé está virado para fora.
- O queixo do bebé toca a mama da mãe.
- A mãe tem 4 dedos abaixo da mama e o dedo grande em cima (mão em "C").
- O bebé não faz barulho a chupar, as bochechas ficam cheias e pode se ouvir o bebé a engolir.

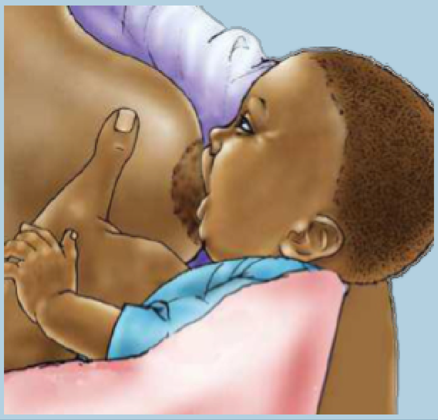

SIM

Elogie à mãe e passe para a próxima página.

NÃO

Demonstre para a mãe os reflexos orais do bebé:

1) Toque nas bochechas (o bebé vira a cabeça em direcção ao toque).

2) Ajude a mãe a estimular o bebé a abrir bem a boca. A mãe pode exprimir algumas gotas de leite para o bebé sentir o cheiro do leite materno e tocar o lábio do bebé com o mamilo.

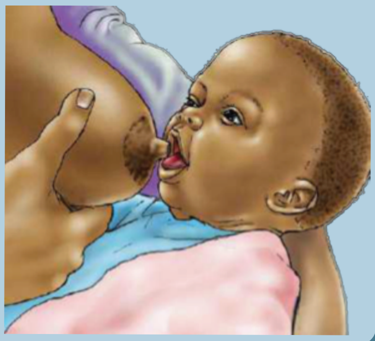

NOTA: Caso o bebé não consiga pegar o peito, refira para a Unidade Sanitária mais próxima.

USAID DO POVO AMERICANO

Maternal and Child Survival Program
Programa de Saúde e Sobrevivência Materna e Infantil

REPÚBLICA DE MOÇAMBIQUE
Ministério da Saúde

MATERIAL DE APOIO PARA PROFISSIONAIS DE SAÚDE QUE APOIAM O ALEITAMENTO MATERNO (PARTE 2)

1 **Pergunte:** O bebé parece ficar satisfeito (larga e mama sozinho, não chora) depois de mamar ?

SIM

Elogie à mãe: Que bom!

Lembre a mãe os sinais de que a amamentação vai bem, ex.: O bebé troca a fralda 6 ou mais vezes por dia, ganha peso a cada mês, atinge os sinais de desenvolvimento para a idade.

Reforce a importância de voltar à unidade sanitária para as consultas de seguimento.

2 **Pergunte:** Há vezes em que o seu peito enche de leite, até doer ?

SIM

Explique:

- Não deixe passar muito tempo entre as mamadas.
- Tire o excesso leite manualmente (ordenha de alívio).
- Ponha panos limpos e quentes nas mamas, ou tome um banho com água morna, antes de tirar o leite.

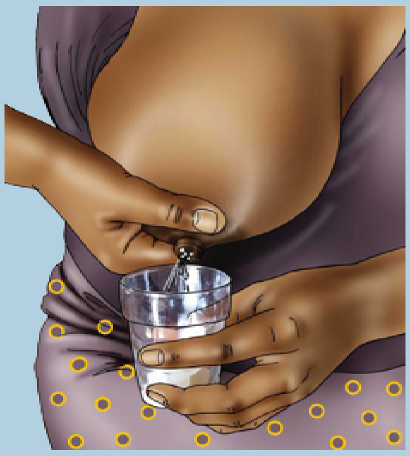

NÃO

Explique: Que bom! Se isto acontecer, procure por mim para eu lhe ajudar.

Sinais de fome:

- Aumenta os movimentos dos olhos fechados ou abertos.
- Abre a boca, estica a língua e vira a cabeça para procurar a mama.
- O bebé fica agitado, mexe os olhos ou faz sons (acordado ou a dormir).
- Chupa ou morde as mãos, dedos, manta ou lençol, ou outro objecto que entra em contacto com a boca.

O choro é o último sinal !

NÃO

Explique que isto pode estar a acontecer porque:

- O bebé não está numa boa posição.
  Verifique o posicionamento (PARTE 1)
- O bebé não está a pegar bem a mama.
  Verifique a boa pega (PARTE 1)
- O bebé não fica o tempo suficiente na mama.
  Aconselhe a dar uma mama de cada vez, até ficar leve e macia. Se o bebé quiser, ofereça a outra mama até ele largar sozinho.

Aconselhe à mãe:

- Amamente muitas vezes durante o dia e a noite (a cada 2-3 horas ou sempre que o bebé quiser) para aumentar a produção de leite.
- Coma regularmente. Aumente a quantidade de comida nas suas refeições e inclua 1-2 lanches entre as refeições.
- Beba muita água limpa e fervida ou tratada com certeza (pelo menos 8 copos por dia).
- Peça apoio da família para fazer as tarefas diárias, cuidar do bebé, e lidar com outras preocupações do dia-a-dia.
- Descanse o suficiente. Aproveite os intervalos em que o bebé descansa e sempre que possível tire uma soneca.

Lembre sempre à mãe que:

- **O leite materno nos primeiros 6 meses é tudo o que o bebé precisa para matar a fome e a sede.**
- **Beber só o leite materno durante os primeiros 6 meses faz crescer e protege.**

REPÚBLICA DE MOÇAMBIQUE
Ministério da Saúde

MATERIAL DE APOIO PARA PROFISSIONAIS DE SAÚDE QUE APOIAM O ALEITAMENTO MATERNO (PARTE 3)

Pergunte: Sente alguma dor nas mamas ou nos mamilos?

→ **NÃO** → **Explique:** Que bom! Se isto acontecer, procure por mim para eu lhe ajudar.

↓ **SIM**

↓ Peça à mãe (e ao marido se for necessário) para observar a mama e / ou a mamada.

Mamilos Doridos

Sinais

- O bebé provavelmente está a abocanhar o mamilo e não a aréola (parte escura da mama), causando dor.

Aconselhamento e Apoio

- Volte para a PARTE 1 e ajude a mãe no posicionamento e pega.
- Aconselhe a continuar a amamentar com boa pega, e a dor irá passar naturalmente.
- Pode extrair e aplicar algumas gotas do leite materno no mamilo para ajudar a diminuir a dor.
- Se a dor não passar naturalmente em 2 dias, volte à Unidade Sanitária.

Rachas ou Feridas nos Mamilos

Sinais

- Poderá ver a pele rachada na ponta ou em torno do mamilo.
- Depois da mamada poderá ver o mamilo achatado e com uma linha branca na ponta.
- O mamilo pode apresentar-se vermelho e ferido.

Aconselhamento e Apoio

Se for HIV-negativa:

- Não interrompa a amamentação. É importante continuar a amamentar com frequência e durante o tempo que o bebé quiser.
- Pode extrair e aplicar algumas gotas do leite materno no mamilo para ajudar a sarar.
- Tire o leite da mama com ferida para evitar que os ductos fiquem obstruidos e recomende que continue a amamentar sempre que o bebé quiser.
- Trate qualquer infecção.
- Se a dor não passar naturalmente em 2 dias, volte à Unidade Sanitária.

Se for HIV-positiva:

- Continue a amamentar sempre que o bebé quiser com a mama boa.
- Pare de amamentar com a mama infectada, a fazer massagens às mamas para retirar o leite para evitar que os ductos fiquem obstruídos.
- Trate qualquer infecção.
- Se a dor não passar naturalmente em 2 dias, volte à Unidade Sanitária.

Ingurgitamento

Sinais

- Uma ou as duas mamas inchadas e com a pele vermelha e brilhante.
- Mamilos achatados e duros, e dificuldade na pega.
- Pode ter febre, que passa em um dia.
- O leite não sai bem.

Aconselhamento e Apoio

- Se o bebé consegue pegar a mama, amamente tantas vezes quanto o bebé quiser em ambos os seios.
- Se o bebé não consegue pegar bem a mama, pressione em torno da aréola para diminuir o inchaço e facilitar a pega.
- Retire o leite com a mão até que as mamas fiquem mais macias. Depois amamente com frequência.
- Aplique panos limpos e quentes nas mamas ou tome um banho com água morna antes de tirar o leite para ajudar a saída do leite.
- Volte à Unidade Sanitária em 48 horas.

Mastite

Sinais

- Há uma área morna ou quente, sensível (dor severa), inchaço numa mama que pode parecer vermelha ou ter estrias avermelhadas.
- A mãe tem febre e / ou calafrios e / ou dores.
- Mau estar geral.

Aconselhamento e Apoio

- Amamente o seu bebé com frequência, e a evite intervalos longos entre as mamadas.
- Aplique panos limpos e quentes sobre a mama afectada e retire o leite se for muito doloroso amamentar.
- Comece por amamentar com a mama afectada (se a dor permitir), se não, comece pela outra para ajudar no fluxo do leite.
- Estimule o fluxo do leite massageando o lado afectado, aplicando panos limpos e quentes, usando soutien não apertado e descansando com o bebé.
- Pode dar ibuprofeno ou paracetamol à mãe para reduzir a inflamação da mama.
- Terapia com antibióticos recomendada se a mãe tem sinais de infecção e febre há mais de 24 horas.
- Volte à Unidade Sanitária em 48 horas para revaliação.

USAID
DO POVO AMERICANO

Maternal and Child Survival Program
Programa de Saúde e Sobrevivência Materna e Infantil
