# Supplementary material for: Strengthening counseling on barriers to exclusive breastfeeding through use of job aids in Nampula, Mozambique
Source: PLoS One. 2019 Dec 2;14(12):e0224939. doi: 10.1371/journal.pone.0224939 (PMC6886792; doi:10.1371/journal.pone.0224939)
Supplement: S3 Fig — (PDF) [file pone.0224939.s003.pdf]

REPÚBLICA DE MOÇAMBIQUE
Ministério da Saúde

MATERIAL DE APOIO PARA TRABALHADORES COMUNITÁRIOS DE SAÚDE QUE APOIAM O ALEITAMENTO MATERNO (PARTE 1)

1 Pergunte: Como está a correr a amamentação?

BEM → MAL

2 Peça: Posso ver como é que o bebé mama?

3 Verifique: BOM POSICIONAMENTO

- O corpo do bebé está direito e virado para o peito da mãe.
- O corpo do bebé está encostado à barriga da mãe (barriga com barriga).
- Todo corpo do bebé está apoiado na mão e braço da mãe.
- A mãe está confortável e não refere dor.

SIM

Elogie à mãe e passe para o ponto 4

Muito bem! É assim mesmo que se põe o bebé a mamar.

NÃO

Verifique a boa pega e passe para o ponto 4

Ajude a mãe a posicionar bem o bebé.

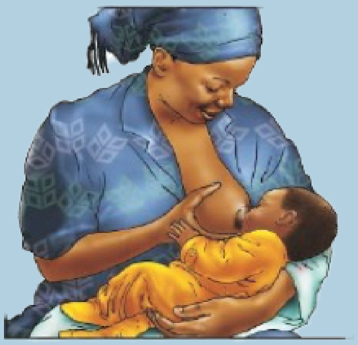

4 Verifique: BOA PEGA

- A boca do bebé está bem aberta.
- O lábio de baixo do bebé está virado para fora.
- O queixo do bebé toca a mama da mãe.
- A mãe tem 4 dedos abaixo da mama e o dedo grande em cima (mão em "C").
- O bebé não faz barulho a chupar, as bochechas ficam cheias e pode se ouvir o bebé a engolir.

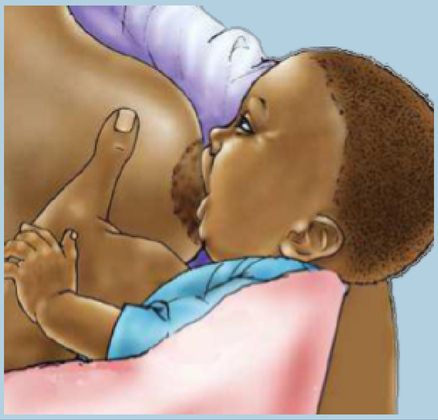

SIM

Elogie à mãe e passe para a próxima página.

NÃO

Demonstre para a mãe os reflexos orais do bebé:

1) Toque nas bochechas (o bebé vira a cabeça em direcção ao toque).

2) Ajude a mãe a estimular o bebé a abrir bem a boca. A mãe pode exprimir algumas gotas de leite para o bebé sentir o cheiro do leite materno e tocar o lábio do bebé com o mamilo.

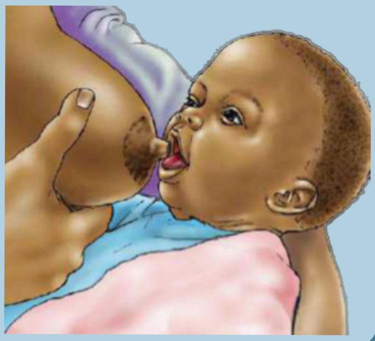

NOTA: Caso o bebé não consiga pegar o peito, refira para a Unidade Sanitária mais próxima.

USAID
DO POVO AMERICANO

Maternal and Child Survival Program
Programa de Saúde e Sobrevivência Materna e Infantil

REPÚBLICA DE MOÇAMBIQUE
Ministério da Saúde

MATERIAL DE APOIO PARA TRABALHADORES COMUNITÁRIOS DE SAÚDE QUE APOIAM O ALEITAMENTO MATERNO (PARTE 2)

1 Pergunte: O bebé parece ficar satisfeito (larga e mama sozinho, não chora) depois de mamar?

SIM

Elogie à mãe: Que bom!

Lembre a mãe os sinais de que a amamentação vai bem, ex.: O bebé troca a fralda 6 ou mais vezes por dia, ganha peso a cada mês, atinge os sinais de desenvolvimento para a idade.

Reforce a importância de voltar à unidade sanitária para as consultas de seguimento.

2 Pergunte: Há vezes em que o seu peito enche de leite, até doer ?

SIM

Explique:

- Não deixe passar muito tempo entre as mamadas.
- Tire o excesso leite manualmente (ordenha de alívio).
- Ponha panos limpos e quentes nas mamas, ou tome um banho com água morna, antes de tirar o leite.

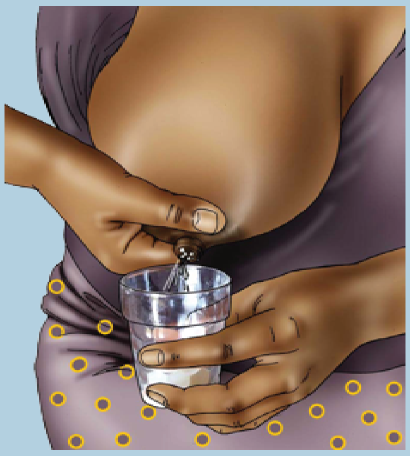

NÃO

Explique: Que bom! Se isto acontecer, procure por mim para eu lhe ajudar.

NÃO

Explique que isto pode estar a acontecer porque:

- O bebé não está numa boa posição. **Verifique o posicionamento (PARTE 1)**
- O bebé não está a pegar bem a mama. **Verifique a boa pega (PARTE 1)**
- O bebé não fica o tempo suficiente na mama. **Aconselhe a dar uma mama de cada vez, até ficar leve e macia. Se o bebé quiser, ofereça a outra mama até ele largar sozinho.**

Aconselhe à mãe:

- Amamente muitas vezes durante o dia e a noite (a cada 2-3 horas ou sempre que o bebé quiser) para aumentar a produção de leite.
- Coma regularmente. Aumente a quantidade de comida nas suas refeições e inclua 1-2 lanches entre as refeições.
- Beba muita água limpa e fervida ou tratada com certeza (pelo menos 8 copos por dia).
- Peça apoio da família para fazer as tarefas diárias, cuidar do bebé, e lidar com outras preocupações do dia-a-dia.
- Descanse o suficiente. Aproveite os intervalos em que o bebé descansa e sempre que possível tire uma soneca.

Sinais de fome:

- Aumenta os movimentos dos olhos fechados ou abertos.
- Abre a boca, estica a língua e vira a cabeça para procurar a mama.
- O bebé fica agitado, mexe os olhos ou faz sons (acordado ou a dormir).
- Chupa ou morde as mãos, dedos, manta ou lençol, ou outro objecto que entra em contacto com a boca.

O choro é o último sinal !

Lembre sempre à mãe que:

- **O leite materno nos primeiros 6 meses é tudo o que o bebé precisa para matar a fome e a sede.**
- **Beber só o leite materno durante os primeiros 6 meses faz crescer e protege.**

REPÚBLICA DE MOÇAMBIQUE
Ministério da Saúde

MATERIAL DE APOIO PARA TRABALHADORES COMUNITÁRIOS DE SAÚDE QUE APOIAM O ALEITAMENTO MATERNO (PARTE 3)

Observe a mamada e Pergunte: Sente alguma dor nas mamas ou nos mamilos ?

SIM

Peça à mãe (e ao marido se for necessário) para observar a mama.

Mamilos Doridos

Sinais

- O bebé provavelmente está a abocanhar o mamilo e não a aréola (parte escura da mama), causando dor.

Aconselhamento e Apoio

- Volte para a PARTE 1 e ajude a mãe no posicionamento e pega.
- Aconselhe a continuar a amamentar com boa pega, e a dor irá passar naturalmente.
- Aconselhe a procurar ajuda novamente se a dor não passar naturalmente em 2 dias.
- Aconselhe a mãe a passar leite materno no mamilo e areola ao final de cada mamada para ajudar a restauração da pele.

NÃO

Explique: Que bom! Se isto acontecer, procure por mim para eu lhe ajudar.

Se encontrar um dos seguintes sintomas:

- Racha na ponta ou em torno do mamilo.
- Mamilo achatado e com uma linha branca na ponta.
- Sangramento nos mamilos.
- Fissuras ou feridas nos mamilos.
- Um caroço numa das mamas.
- Uma zona mais quente, sensível, vermelha numa mama.
- Mãe com febre e / ou tremor e / ou dores mesmo que aparentemente não tenha relação com as mamas.

REFIRA PARA A UNIDADE SANITÁRIA MAIS PRÓXIMA.
Sensibilize a mãe a pedir a contra-referência ao técnico de saúde.

USAID
DO POVO AMERICANO

Maternal and Child Survival Program
Programa de Saúde e Sobrevivência Materna e Infantil
